# AXL Expression on Homeostatic Resident Liver Macrophages Is Reduced in Cirrhosis Following GAS6 Production by Hepatic Stellate Cells

**DOI:** 10.1016/j.jcmgh.2023.03.007

**Published:** 2023-03-31

**Authors:** Oltin-Tiberiu Pop, Anne Geng, Emilio Flint, Arjuna Singanayagam, Caner Ercan, Lucia Possamai, Vishal C. Patel, Patrizia Kuenzler, Marie-Anne Meier, Savas Soysal, Petr Hruz, Otto Kollmar, Kate C. Tatham, Josie K. Ward, Beat Müllhaupt, Achim Weber, Julia Wendon, Jan Hendrik Niess, Markus Heim, David Semela, Christopher Weston, Charalambos G. Antoniades, Luigi Maria Terracciano, Evangelos Triantafyllou, Robert G. Brenig, Christine Bernsmeier

**Affiliations:** 1 Medical Research Centre and Division of Gastroenterology and Hepatology, Cantonal Hospital St Gallen, St Gallen, Switzerland; 2 Institute of Immunobiology, Medical Research Centre, Cantonal Hospital St Gallen, St Gallen, Switzerland; 3 Department of Biomedicine, University of Basel, Basel, Switzerland; 4 Institute of Liver Studies, King’s College Hospital, King’s College London, London, United Kingdom; 5 Hepatology Department, St Mary’s Hospital, Imperial College London, London, United Kingdom; 6 University Hospital, Basel, Institute of Pathology, Basel, Switzerland; 7 The Roger Williams Institute of Hepatology London, Foundation for Liver Research, London, United Kingdom & School of Immunology and Microbial Sciences, Faculty of Life Sciences and Medicine, King’s College London, London, United Kingdom; 8 Gastroenterology and Hepatology, University Centre for Gastrointestinal and Liver Diseases, Basel, Switzerland; 9 Department of Visceral Surgery, University Centre for Gastrointestinal and Liver Diseases, Basel, Switzerland; 10 Section of Anaesthetics, Pain Medicine and Intensive Care Medicine, Department of Surgery and Cancer, Imperial College London and Imperial College Healthcare NHS Trust, London, United Kingdom; 11 Department of Gastroenterology and Hepatology, University Hospital Zurich, Zurich, Switzerland; 12 Department of Pathology and Molecular Pathology, University Hospital Zurich, Zurich, Switzerland; 13 Centre for Liver Research and National Institute for Health Research, Biomedical Research Unit, University of Birmingham, Birmingham, United Kingdom

**Keywords:** TAM Receptors, Innate Immunity, Cirrhosis, Resident Liver Macrophages

## Abstract

**Background & Aims:**

AXL and MERTK expression on circulating monocytes modulated immune responses in patients with cirrhosis (CD14^+^HLA-DR^+^AXL^+^) and acute-on-chronic liver failure (CD14^+^MERTK^+^). AXL expression involved enhanced efferocytosis, sustained phagocytosis, but reduced tumor necrosis factor-α/interleukin-6 production and T-cell activation, suggesting a homeostatic function. Axl was expressed on murine airway in tissues contacting the external environment, but not interstitial lung- and tissue-resident synovial lining macrophages. Here, we assessed AXL expression on tissue macrophages in patients with cirrhosis.

**Methods:**

Using multiplexed immunofluorescence we compared AXL expression in liver biopsies in cirrhosis (n = 22), chronic liver disease (n = 8), non-cirrhotic portal hypertension (n = 4), and healthy controls (n = 4). Phenotype and function of isolated primary human liver macrophages were characterized by flow cytometry (cirrhosis, n = 11; control, n = 14) *ex vivo*. Also, AXL expression was assessed on peritoneal (n = 29) and gut macrophages (n = 16) from cirrhotic patients. Regulation of AXL expression was analyzed *in vitro* and *ex vivo* using primary hepatic stellate cells (HSCs), LX-2 cells, and GAS6 in co-culture experiments.

**Results:**

AXL was expressed on resident (CD68^+^) but not tissue-infiltrating (MAC387^+^) liver macrophages, hepatocytes, HSCs, or sinusoidal endothelial cells. Prevalence of hepatic CD68^+^AXL^+^ cells significantly decreased with cirrhosis progression: (healthy, 90.2%; Child-Pugh A, 76.1%; Child-Pugh B, 64.5%; and Child-Pugh C, 18.7%; all *P* < .05) and negatively correlated with Model for End-Stage Liver Disease and C-reactive protein (all *P* < .05). AXL-expressing hepatic macrophages were CD68^high^HLA-DR^high^CD16^high^CD206^high^. AXL expression also decreased on gut and peritoneal macrophages from cirrhotic patients but increased in regional lymph nodes. GAS6, enriched in the cirrhotic liver, appeared to be secreted by HSCs and down-regulate AXL *in vitro*.

**Conclusions:**

Decreased AXL expression on resident liver macrophages in advanced cirrhosis, potentially in response to activated HSC-secreted GAS6, suggests a role for AXL in the regulation of hepatic immune homeostasis.

## Graphical abstract

**Figure:**
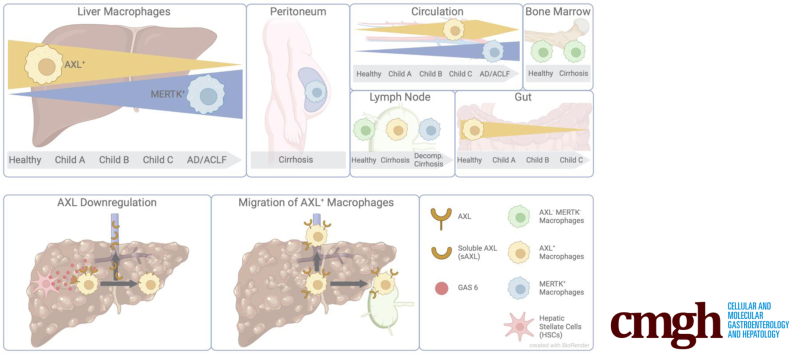

**Summary**

Enhanced AXL expression on circulating monocytes attenuated immune responses in cirrhosis patients. Resident liver macrophages highly express AXL physiologically. GAS6, secreted by activated hepatic stellate cells, decreased AXL on liver macrophages in relation to cirrhosis progression. This suggests a role for AXL in the regulation of hepatic immune homeostasis.

Under homeostatic conditions, the liver is a tolerogenic organ acting as a firewall mediating mutualism between the host and its gut commensal microbiota.^1^ Hepatic non-parenchymal cells including resident liver macrophages, dendritic cells (DCs), infiltrating monocytes, hepatic stellate cells (HSCs), and liver sinusoidal endothelial cells actively promote tolerance by removing bacterial products, secreting tolerogenic factors, and modulating immune cell phenotype and function.^1^^,^^2^ During the progression of cirrhosis and evolution of portal hypertension, these mechanisms fail to maintain tolerance, which has pivotal consequences for the hepatic and systemic milieu.^3^ Also, a dysfunctional intestinal barrier resulting in pathologic bacterial translocation^4^ and altered peritoneal macrophages (pMACs), which are critical for regulating inflammation and controlling bacterial infections,^5^ contribute to the loss of systemic immune surveillance during development of cirrhosis. The current pathophysiological concept assumes that the breakdown of these “immunologic firewalls” in tissues in contact with the external environment predisposes to the development of bacterial infections, sepsis, organ dysfunction, progressive hepatocellular failure, and accelerates morbidity and mortality.^6^, ^7^, ^8^

TYRO-3, AXL, and MERTK belong to the TAM receptor tyrosine kinases family and are expressed on monocytes and macrophages. They are important regulators of innate immune homeostasis^9^^,^^10^ and operate by inhibiting toll-like receptor (TLR) signaling pathways and promoting phagocytic removal of apoptotic cells.^9^^,^^11^

The function of AXL-expressing innate immune cells in tissues acting as immunologic barriers has been widely addressed in distinct compartments such as the lung,^12^ central nervous system,^13^^,^^14^ joints,^15^ and skin^16^ and associated with prevention and resolution of inflammation. In mouse livers, recent analyses indicated that Mertk and Axl were predominantly expressed on Kupffer cells (KCs) and endothelial cells and critical regulators to maintain liver homeostasis.^17^ In patients with acutely decompensated cirrhosis and especially those with acute-on-chronic liver failure, MERTK-expressing macrophages were detected in the liver and other tissue sites such as the peritoneum and regional lymph nodes.^18^ MERTK-expressing monocytes and macrophages represented an immune-modulatory pro-restorative cell population, characterized by enhanced efferocytosis of apoptotic neutrophils while expressing attenuated cytokine responses.^18^^,^^19^ Recently, we observed circulating AXL-expressing monocytes in patients with cirrhosis of the liver in parallel with disease progression and portal hypertension characterized by preserved phagocytosis capacity but reduced T-cell activation and inflammatory cytokines suggesting a homeostatic function.^20^

Considering cirrhosis as a systemic disease condition, in this study we sought to assess the plasticity of AXL expression on tissue macrophages in different compartments of patients with advanced cirrhosis and to investigate its potential role in immune homeostasis regulation.

## Results

### Patient Characteristics

The 2 cohorts comprising patients with cirrhosis distinguished between Child-Pugh A, B, and C and compared with healthy controls and chronic liver disease without cirrhosis, and patients with cirrhosis undergoing liver resection compared with histologically normal controls are summarized in Tables 1 and 2. Both cohorts were characterized by etiology, disease severity scores, and extensive clinical parameters. In the first cohort in patients with cirrhosis, 1-year mortality rate was 13.6% (n = 3) (hepatocellular carcinoma 33% [n = 1], sepsis 33% [n = 1], and acute respiratory distress syndrome 33% [n = 1]), and liver transplantation occurred in 13.6% (n = 3). Infections at time of inclusion were seen in 9% of patients (urinary tract [n = 2]), whereas 18.2% (n = 4) (spontaneous bacterial peritonitis 50% [n = 2], hospital-acquired pneumonia 25% [n = 1], sepsis of unknown origin 25% [n = 1]) of patients with cirrhosis developed infections within 15 months after inclusion. Of note, one patient presented with infection both at time of inclusion and after inclusion. In the second cohort, 1-year mortality in patients with cirrhosis was 27.2% (hepatocellular carcinoma [n = 3]).

**Table 1:** Clinical Characteristics of Patients With Cirrhosis Compared with Chronic Liver Disease Without Cirrhosis and Healthy Controls

Variables, median (range)	Cirrhosis (n = 22)		CLD without cirrhosis (n = 8)		Healthy controls (n = 4)
Age (*y*)	59 (34–79)		38 (24–66)^a^		55 (47–69)
Sex (m:f)	14:8		5:3		—
Underlying liver disease(s) [%]	ALD HCV NAFLD PBC Wilson’s disease Hemochromatosis Unknown	11 [50] 4 [18] 2 [9] 2 [9] 1 [5] 1 [5] 1 [5]	HBV NAFLD AIH	4 [50] 3 [37.5] 1 [12.5]	NA
Death (1 year)	3 [14]		—		—
Child-Pugh	8 (5–11)		5 (5–5)		NA
MELD	14 (6–29)		6 (6–6)		NA
BMI (kg/m^2^)	27 (17–32)		—		—
Na^+^ (mmol/L)	138 (120–144)		139 (137–142)		140 (138–142)
K^+^ (mmol/L)	3.9 (3.0–5.1)		3.8 (3.7–4.0)		3.95 (3.40–4.20)
Creatinin (μmol/L)	84 (47–171)		70.5 (61.0–83.0)		83.5 (73.0–93.0)
Urea (mmol/L)	5.9 (3.1–14.7)		3.7 (3.5–3.9)		6.75 (4.8–7.6)
Bilirubin (μmol/L)	34 (8–147)		16 (5–32)		9.5 (6–12)^b^
AST (U/L)	49 (16–168)		28 (17–133)		23 (22–27)^b^
ALT (U/L)	29.5 (7.0–114.0)		27 (11–207)		30.5 (15.0–35.0)
GGT (U/L)	85 (26–1013)		26 (10–272)		119 (20–133)
AP (U/L)	117 (49–483)		76 (35–106)		56 (33–93)^c^
INR	1.45 (0.90–2.30)		1 (1–1)^a^		—
Albumin (g/L)	26.5 (19.0–39.0)		39.8 (37.0–44.4)^d^		39 (38–40)^e^
Hemoglobin (g/L)	99.5 (7.2–152.0)		142 (113–164)^d^		—
Hematocrit (%)	0.314 (0.242–0.440)		0.42 (0.35–0.5)^a^		—
Leukocytes (G/L)	5.05 (2.40–70.0)		5.4 (4.3–6)		—
Neutrophils (G/L)	3.6 (1.6–41.3)		48.9 (48.6–55.1)^f^		—
Eosinophils (G/L)	0.29 (0.017–0.91)		3.9 (2.2–5.7)		—
Basophils (G/L)	0.37 (0.001–0.28)		0.7 (0.6–1.3)		—
Lymphocytes (G/L)	1.55 (0.4–2.5)		2.3 (1.6–2.5)		—
Monocytes (G/L)	0.62 (0.23–7.49)		5.8 (5.7–7.3)		—
Platelets (G/L)	100.5 (8.5–341)		218 (172–235)^a^		—
CRP (mg/L)	9 (0.4–67)		2 (0.6–5.4)^f^		1.35 (0.70–2.40)^b^

NOTE. Data indicated as median with (minimum and maximum) and [percentage].

AIH, autoimmune hepatitis; ALD, alcoholic liver disease; ALT, alanine aminotransferase; AP, alkaline phosphatase; AST, aspartate aminotransferase; BMI, body mass index; CRP, C-reactive protein; GGT, gamma-glutamyl transferase; HBV, hepatitis B virus; HCV, hepatitis C virus; INR, international normalized ratio; MELD, Model for End-Stage Liver Disease; NAFLD, nonalcoholic fatty liver disease; PBC, primary biliary cirrhosis.

a *P* = .01.

b *P* = .01.

c *P* = .05.

d *P* = .001 indicate cirrhosis vs CLD without cirrhosis.

e *P* = .001 indicate cirrhosis vs healthy controls; comparisons by Mann-Whitney *U* tests.

f *P* = .05.

**Table 2:** Clinical Characteristics of Patients With Cirrhosis, Pathologic Controls, and Histologically Normal Controls

Variables, median (range)	Cirrhosis (n = 11)		Pathologic controls (n = 3)		Histologically normal controls (n = 12)	
Age (*y*)	67 (48–84)		71 (64–78)		61 (28–78)	
Sex (m:f)	6:5		2:1		5:10	
Underlying liver disease(s) [%]	ALD HCV AIH Unknown	6 [54] 3 [27] 1 [9] 1 [9]	NA Echinococcosis	2 [66] 1 [33]	NA Echinococcosis	11 [79] 3 [21]
Death (1 year)	3 [27]		0 [0]		0 [0]	
Child-Pugh	A (n = 9), B (n = 2)		NA		NA	
MELD	8 (6–15)		NA		NA	
BMI (kg/m^2^)	27 (22–34)		23 (21–25)		23 (20–27)	
Na^+^ (mmol/L)	139 (132–143)		140 (140–141)		140 (136–141)	
K^+^ (mmol/L)	4 (3.7–4.7)		4.1 (3.8–4.4)		4.3 (3.8–4.5)	
Ca^2+^ (mmol/L)	2.4 (2.1–2.6)		2.4 (2.3–2.5)		2.4 (2.2–2.5)	
Creatinin (μmol/L)	63 (48–132)		79 (61–100)		58 (42–89)	
GFR	92 (51–109)		80 (62–92)		97 (66–127)	
Urea (mmol/L)	5.7 (2.6–10.7)		6.5 (4.6–8.6)		4.9 (3.8–8.4)	
Bilirubin (μmol/L)	10.5 (4.6–19.6)^a^		5.8 (4.1–8.8)		5.1 (2.5–14.2)	
AST (U/L)	33 (20–83)		30 (24–36)		31 (18–52)	
ALT (U/L)	26 (13–48)		24 (14–29)		30 (16–56)	
GGT (U/L)	70 (18–796)		316 (31–858)		57 (16–576)	
AP (U/L)	80 (51–557)		221 (67–487)		95 (44–354)	
INR	1.1 (1.0–1.9)^a^		1 (0.9–1.0)		1 (0.9–1.1)	
Albumin (g/L)	38 (25–43)		37 (35–42)		36 (29–41)	
Hemoglobin (g/L)	134 (88–143)		126 (107–155)		119 (104–152)	
Hematocrit (%)	40 (28–43)		36 (31–43)		36 (31–43)	
Leukocytes (G/L)	5.65 (2.74–8.67)		6.00 (4.98–7.05)		5.4 (3.1–12.0)	
Erythrocytes (G/L)	4.3 (3.03–5.17)		4.14 (3.67–5.01)		4.12 (3.64–4.98)	
Neutrophils (G/L)	2.66 (1.97–6.23)		3.94 (2.91–4.91)		3.26 (2.02–4.85)	
Eosinophils (G/L)	0.07 (0.05–0.16)		0.14 (0.03–0.32)		0.12 (0.04–0.59)	
Basophils (G/L)	0.02 (0.01–0.03)		0.03 (0.02–0.04)		0.02 (0.01–0.08)	
Lymphocytes (G/L)	1.02 (0.48–1.85)		1.42 (1.2–1.65)		1.46 (1.05–2.37)	
Monocytes (G/L)	0.31 (0.19–0.61)		0.40 (0.30–0.46)		0.33 (0.22–1)	
Platelets (G/L)	137 (99–279)^a^		238 (211–264)		242 (100–363)	
CRP (mg/L)	3.4 (0.5–74.2)		7.3 (0.5–10.8)		2.35 (0.7–13.2)	

NOTE. Data indicated as median with (minimum and maximum) and [percentage].

AIH, autoimmune hepatitis; ALD, alcoholic liver disease; ALT, alanine aminotransferase; AP, alkaline phosphatase; AST, aspartate aminotransferase; BMI, body mass index; CRP, C-reactive protein; GFR, glomerular filtration rate; GGT, gamma-glutamyl transferase; HCV, hepatitis C virus; INR, international normalized ratio; MELD, Model for End-Stage Liver Disease.

a *P* = .05 indicates comparison between cirrhosis and histologically normal controls by Mann-Whitney *U* test.

### AXL Is Expressed on the Majority of Resident Human Liver Macrophages

**Figure 1:**
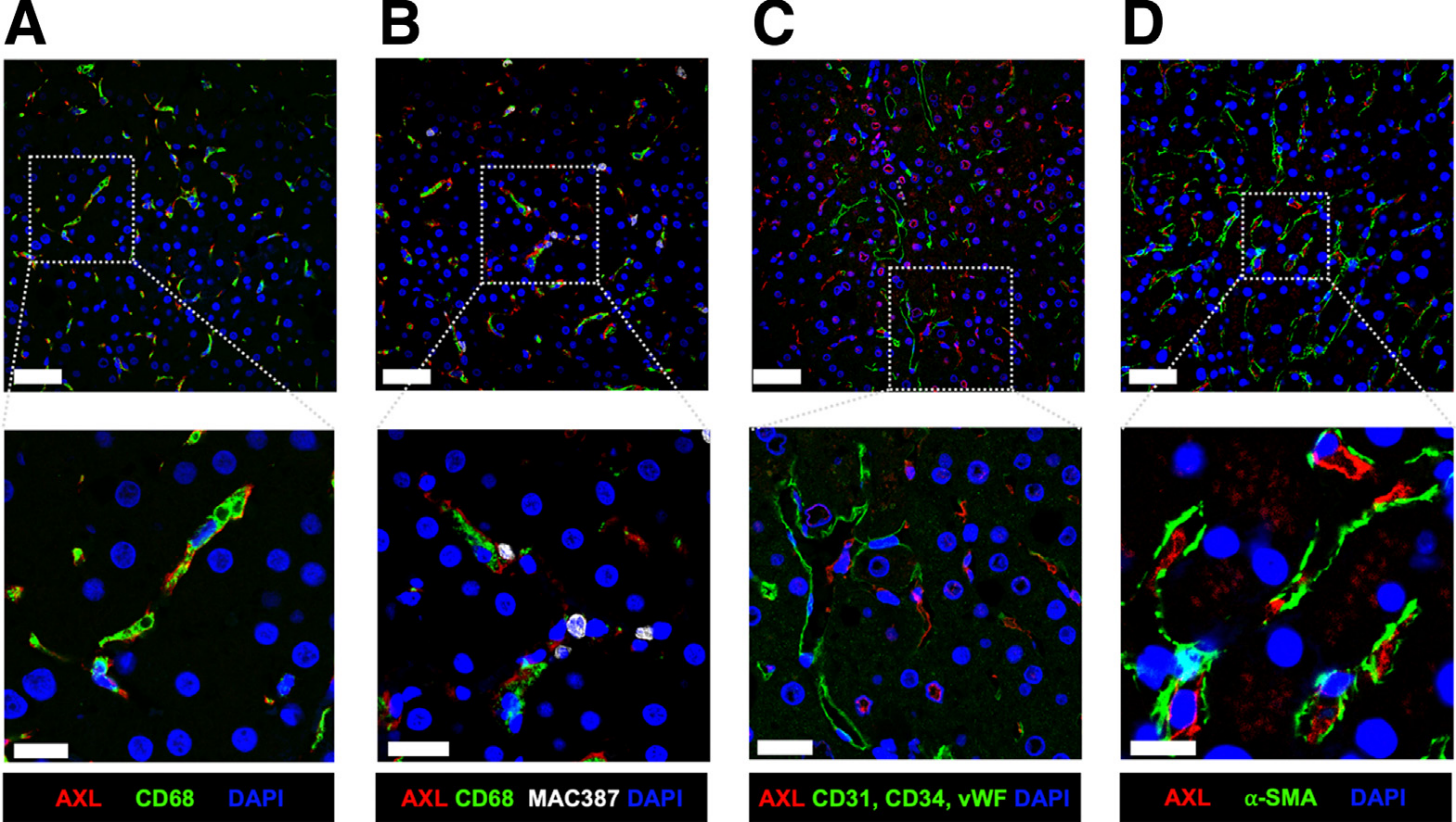
**AXL is expressed on resident liver macrophages.** Representative micrographs from immunofluorescence stains of liver biopsies from the control group (n = 4). (*A*) AXL/CD68/DAPI stain showing AXL expression on resident liver macrophages. (*B*) AXL/CD68/MAC387/DAPI stain showing absence of AXL expression on liver-infiltrating macrophages. (*C*) AXL/CD31+CD34+vWF/DAPI stain showing absence of AXL expression on LSECs. (*D*) AXL/α-SMA/DAPI stain of a control liver biopsy showing absence of AXL expression on aHSCs. Upper panels: original magnification, 400×; scale bar = 50 μm; lower panels: details, scale bar = 20 μm. AF594, *red*; AF488, *green*; AF647, *white*; DAPI, *blue*.

**Figure 2:**
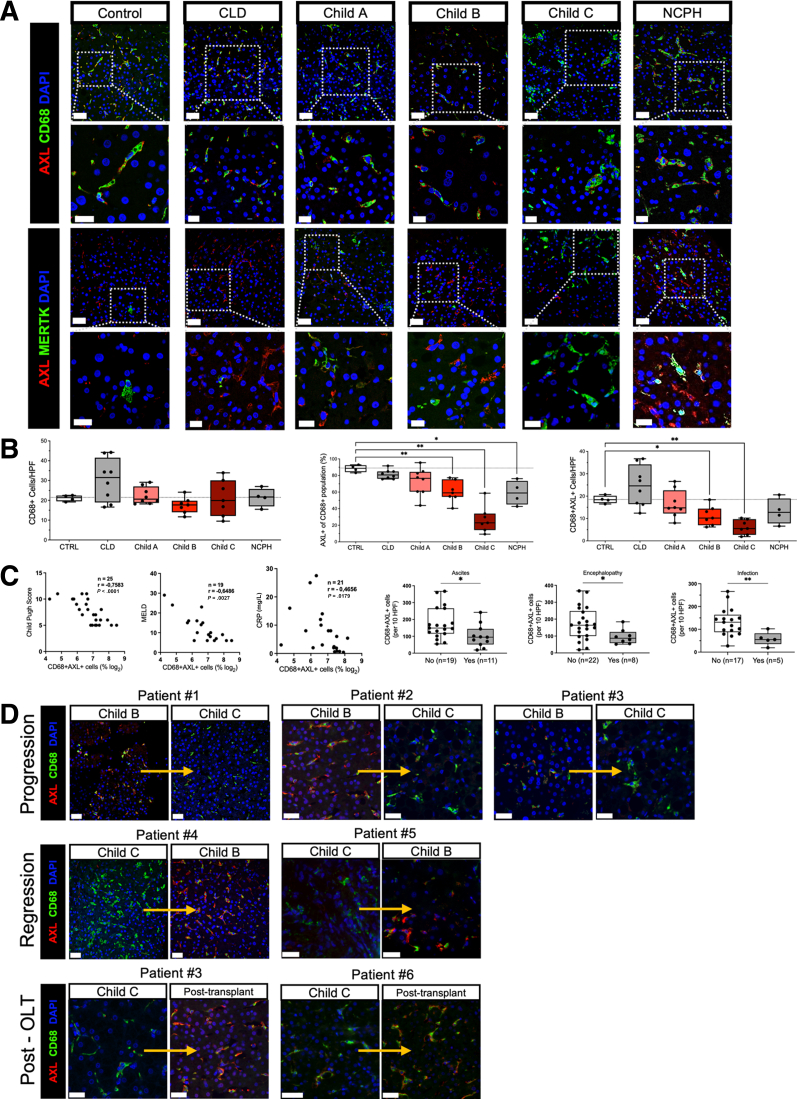
**Loss of AXL-expressing resident liver macrophages with the progression of cirrhosis.** (*A*) Representative immunofluorescence micrographs from AXL/CD68/DAPI and AXL/MERTK/DAPI stains of liver biopsies from the control group (n = 4), chronic liver disease (CLD) (n = 8), Child-Pugh A (n = 8), Child-Pugh B (n = 7), Child-Pugh C (n = 7), and NCPH (n = 4). Upper panels: original magnification, 400×; scale bar = 50 μm; lower panels: details, scale bar = 20 μm. (*B*) Cell count of CD68^+^ resident liver macrophages per HPF, AXL^+^ macrophages per HPF, and percentage of AXL^+^ cells of CD68^+^ population. (*C*) Correlations of AXL^+^ macrophages with Child-Pugh and MELD scores, C-reactive protein, encephalopathy, ascites, and infections. ∗*P* ≤ .05/∗∗*P* ≤ .01 (Mann-Whitney tests, Spearman correlation coefficients). (*D*) AXL/CD68/DAPI stain displaying longitudinal AXL expression on resident liver macrophages from patients undergoing either progression or regression or resolution of cirrhosis post transplantation (OLT). AF488, *green*; AF647, *red*; DAPI, *blue*; scale bar = 50 μm.

Using immunofluorescence microscopy, we investigated AXL expression on hepatocytes, resident macrophages (CD68^+^, Figure 1*A*), recently infiltrated macrophages (MAC387^+^,^21^ Figure 1*B*), endothelial cells (CD31/CD34/vWF^+^, Figure 1*C*), and activated HSCs (aHSCs) (alpha smooth muscle actin [α-SMA^+^],^22^ Figure 1*D*). We hereby identified AXL expression on the majority of resident macrophages in normal liver tissue but not on hepatocytes or other non-parenchymal cells. Recently infiltrated macrophages were sparse in healthy liver tissue and barely expressed AXL (Figure 1). Co-expression of the TAM receptors Mertk and Axl had been described on freshly isolated murine KCs.^17^ In our study on human liver tissue, we did not observe AXL and MERTK co-localization (Figure 2*A*).

### AXL Expression on Macrophages Is Reduced in Patients With Advanced Cirrhosis and Portal Hypertension

Having observed AXL expression on most resident macrophages in healthy livers, we assessed AXL expression at different disease stages of cirrhosis (Figure 2*A*). Although CD68^+^ macrophages did not differ significantly across different stages of cirrhosis, the number of AXL-expressing CD68^+^ resident liver macrophages per high-power field (HPF) was significantly reduced in advanced stages of cirrhosis, as was the percentage of AXL^+^ cells relative to the CD68^+^ macrophage population (Figure 2*B*). Similarly, AXL-expressing macrophages were reduced in non-cirrhotic portal hypertension (NCPH) (Figure 2*A* and *B*). As shown previously,^18^^,^^19^ MERTK-expressing cells were increased in patients with advanced cirrhosis compared with controls (Figure 2*A*).

AXL expression on liver macrophages correlated negatively with disease severity scores (Child-Pugh and Model of End-Stage Liver Disease [MELD] scores) and a marker of inflammation (C-reactive protein) (Figure 2*C*). Furthermore, presence of ascites, hepatic encephalopathy, and infectious complications were associated with low numbers of CD68^+^AXL^+^ cells (Figure 2*C*).

In 6 patients we assessed AXL expression longitudinally. These patients clinically showed either progression from early to advanced stages of cirrhosis and underwent follow-up biopsies or transplantation, respectively, or regression of their initially advanced cirrhosis stage. A different condition studied was resolution of cirrhosis in those patients who underwent transplantation. We found a decrease in AXL^+^ liver macrophages in parallel with progression of cirrhosis stage (Figure 2*D*, patients 1–3). Inversely, we observed an increase of AXL^+^ macrophages along with cirrhosis regression (Figure 2*D*, patients 4 and 5) or after liver transplantation (Figure 2*D*, patients 3 and 6). Altogether, the data suggest dynamic changes of resident macrophage states during disease evolution.

### AXL and MERTK Expression in Areas of Fibrosis

**Figure 3:**
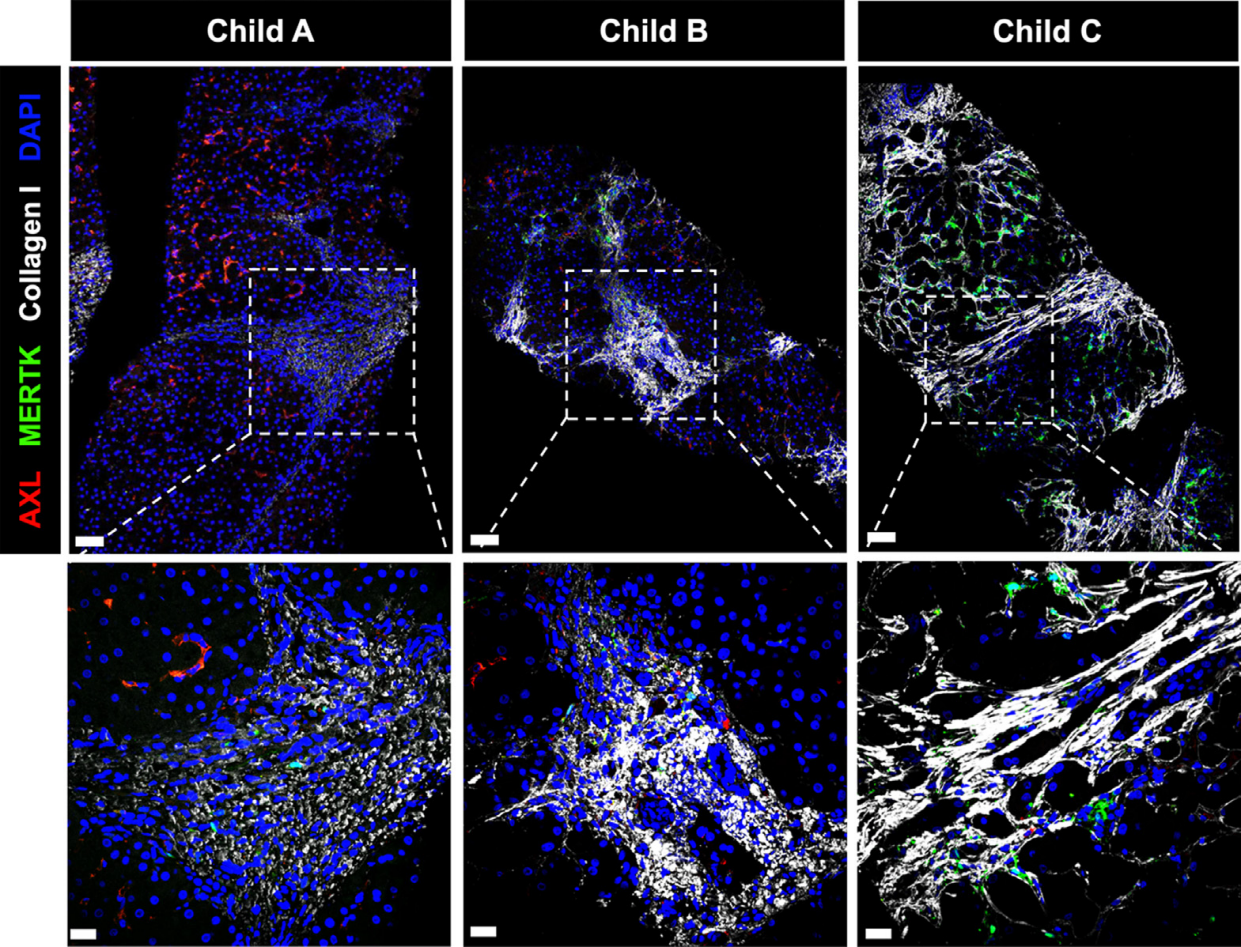
**Reduced AXL expression in portal-septal areas.** Representative micrographs from immunofluorescence stains of liver biopsies from patients with Child-Pugh A (*left*), Child-Pugh B (*middle*), and Child-Pugh C (*right*) cirrhosis. AXL/MERTK/collagen I/DAPI stain showing AXL and MERTK expression in relation to fibrotic areas; FITC, *green*; AF594, *red*; AF647, *white*; DAPI, *blue*; scale bar in upper panels = 50 μm, scale bar in lower panels = 20 μm.

Although we performed quantification of the non-parenchymal cells in hepatic panels, this was challenging to describe in areas of fibrosis because of their significant anatomic variability. Immunofluorescent staining for AXL/MERTK/collagen I/DAPI revealed no expression of AXL in the areas of fibrosis, whereas MERTK-expressing cells seem to approximate collagen I deposition in stages Child-Pugh B and C (Figure 3).

### Phenotypic Characterization of the AXL-Expressing Hepatic Macrophages

**Figure 4:**
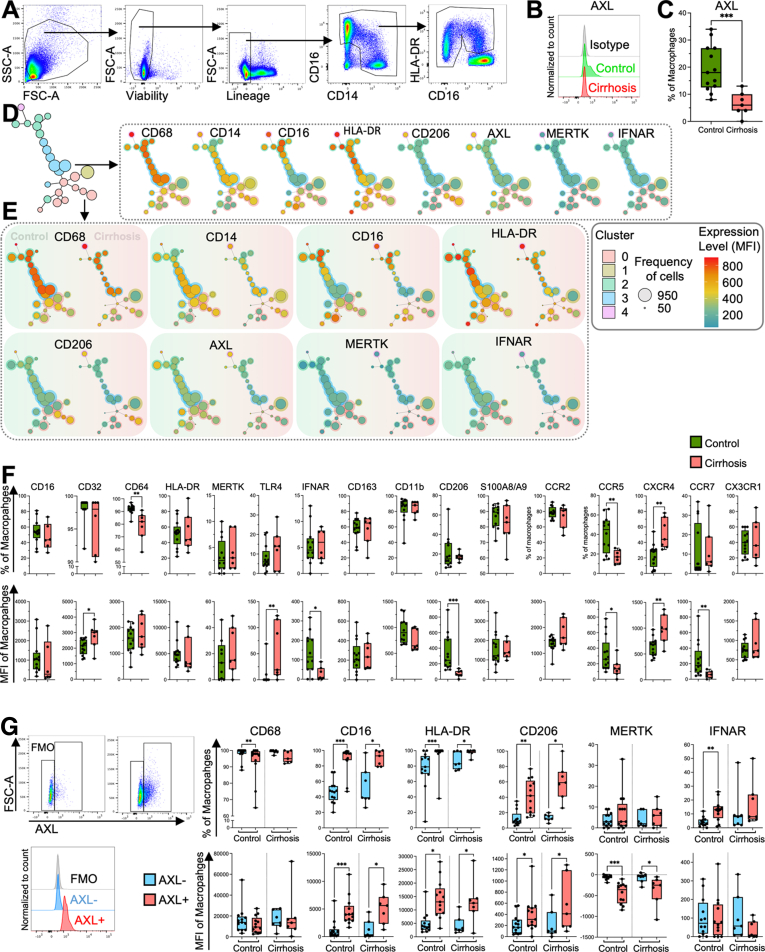
**Immunophenotyping by flow cytometry of liver macrophages.** (*A*) Gating strategy with representative flow cytometry scatter plots. Side scatter area (SSC-A), forward scatter area (FSC-A). (*B*) Representative histograms for AXL expression on liver macrophages in control and compensated cirrhosis. (*C*) AXL expression on liver macrophages (%) in controls (n = 13) and compensated cirrhosis (n = 7). *Box plots* showing median with 10–90 percentile and all points min-max. ∗∗∗*P* ≤ .001 (Mann-Whitney test). (*D*) Scaffold reference map of the mixed control (n = 5, 2550 cells/sample) and compensated cirrhosis (n = 5, 2550 cells/sample) macrophages landscape constructed from fluorescence cytometry data displaying 25 unsupervised FlowSOM nodes in 5 clusters with representative fluorescent marker expression in the scaffold map. (*E*) Comparison of control (n = 5, 2550 cells/sample) and compensated cirrhosis (n = 5, 2550 cells/sample) macrophages representative fluorescent marker expression mapped in the scaffold map. (*F*) Immunophenotyping of control and compensated cirrhosis liver macrophages. Expression level as percentage of all macrophages and as median fluorescence intensity (MFI) of all macrophages. (*G*) Gating strategy and histograms for AXL expression used to distinguish AXL-expressing from AXL-negative liver macrophages. Immunophenotyping of AXL^+^ and AXL^–^ liver macrophages in controls (n = 13) and compensated cirrhosis (n = 7). Expression level as percentage (%) and MFI of all macrophages. *Box plots* showing median with 10–90 percentile and all points min-max. ∗*P* ≤ .05/∗∗*P* ≤ .01/∗∗∗*P* ≤ .001, Mann-Whitney and Wilcoxon test.

In addition to immunofluorescence, we used flow cytometry to characterize the phenotype and function of primary human macrophages obtained from liver resections (Figure 4*A*). In line with our microscopic findings, we found significantly fewer AXL-expressing macrophages in cirrhotic livers when compared with controls (Figure 4*B* and *C*). Using FlowSOM, liver macrophages were split into 5 main clusters and annotated on the tree map (Figure 4*D*). High CD68 expression in clusters 2, 3, and 4 indicated resident liver macrophages (Figure 4*D*), whereas low CD68 in clusters 0 and 1, mainly present in cirrhosis samples, indicated infiltrating macrophages (Figure 4*E*). AXL-expressing macrophages were found in most of the clusters, with AXL^hi^ in clusters 2, 3, and 4 (Figure 4*D*). In contrast, MERTK expression was low in all clusters and allocated to cluster 1, confirming that AXL and MERTK were not co-expressed (Figure 4*D*). Also, a decrease of AXL expression was seen in all clusters, whereas MERTK-expressing macrophages increased slightly in cirrhosis cluster 1 (Figure 4*E*).

We reported higher proportions of CXCR4-expressing and lower proportions of CD64- and CCR5-expressing macrophages within the entire resident macrophage population in cirrhosis (Figure 4*F*, upper panel). The expression of CD32, TLR4, and CXCR4 was increased in cirrhosis, whereas the expression of IFNAR, CD206, CCR5, and CCR7 was reduced (Figure 4*F*, lower panel). AXL-expressing macrophages were identified as CD16^high^HLA-DR^high^, indicating a mature macrophage population (Figure 4*G*). In addition, AXL-expressing macrophages expressed higher levels of mannose receptor CD206 and TAM co-receptor interferon α/beta-receptor (IFNAR, Figure 4*G*).

### AXL-Expressing Hepatic Macrophages Reveal Unaltered Phagocytosis Capacity

**Figure 5:**
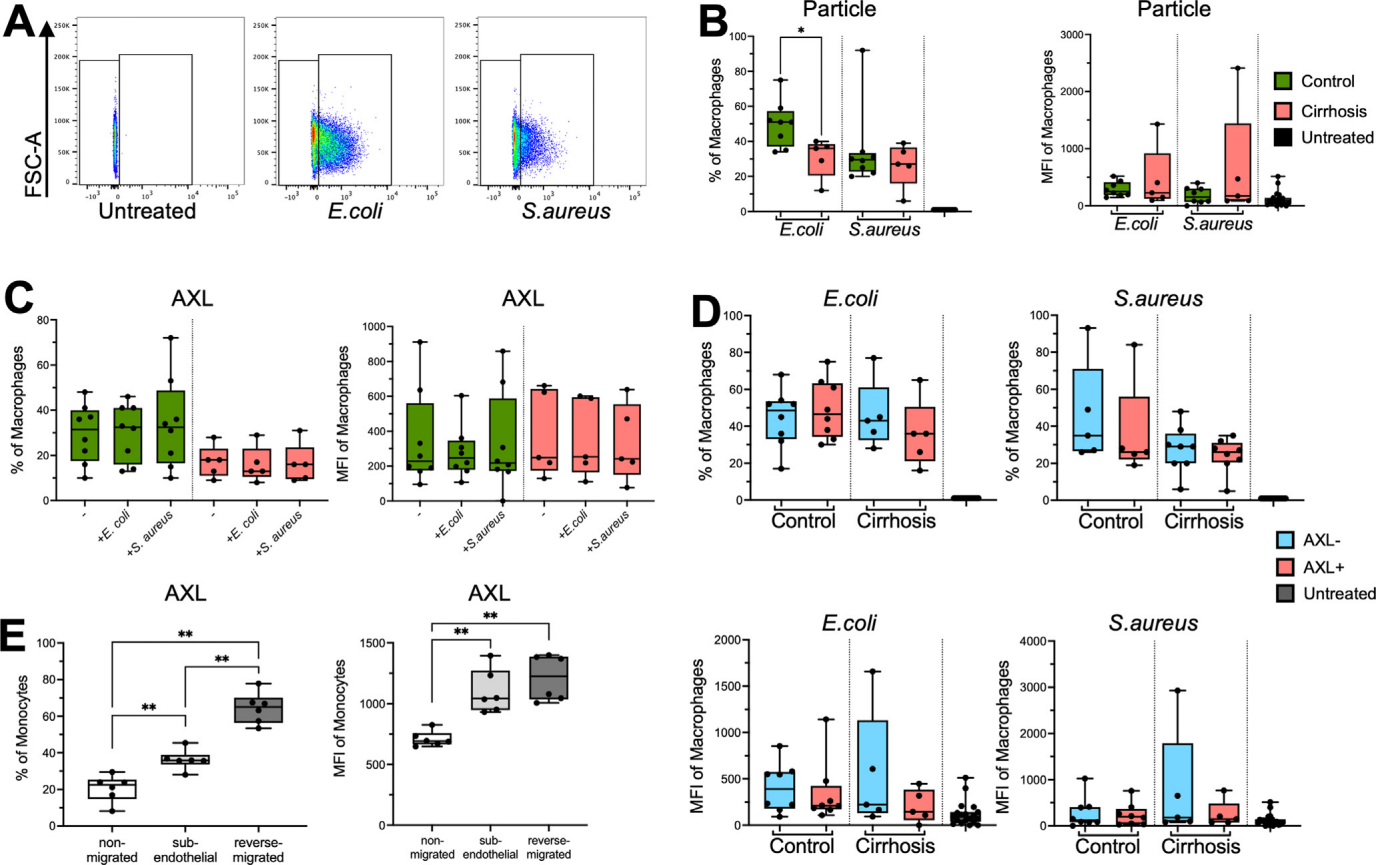
**Phagocytosis of bacteria by primary liver macrophages and AXL on migrating monocyte.** (*A*) Gating strategy for determination of bioparticle (*E coli, S aureus*) positive macrophages from liver resections based on 1% border of untreated control. FSC-A (forward scatter area). (*B*) *E coli* and *S aureus* bioparticle uptake by primary liver macrophages from controls (n = 8) and compensated cirrhosis samples (n = 5); untreated control (n = 8). In percentage and median fluorescence intensity (MFI) of macrophages. (*C*) AXL expression in percentage and MFI on *E coli* and *S aureus* bioparticle uptake on macrophages from controls (n = 8), compensated cirrhosis (n = 5), and untreated controls (n = 8). (*D*) Phagocytosis capacity for *E coli* and *S aureus* bioparticles of AXL^+^/AXL^-^ liver macrophages in percentage and MFI of macrophages from control (n = 8) and compensated cirrhosis samples (n = 5) and untreated controls (n = 8). (*E*) AXL expression levels as percentage and MFI on magnetically sorted CD14^+^ monocytes from healthy donors in an established migration assay (n = 6). *Box plots* showing median with 10–90 percentile and all points min-max. ∗*P* ≤ .05, ∗∗*P* ≤ .01, Wilcoxon, Kruskal-Wallis, and Mann-Whitney test.

Phagocytosis of resident liver macrophages is crucial to maintain tolerance at the barrier between the portal and the systemic circulation. We thus investigated *ex vivo* phagocytosis capacity of resident macrophages from compensated cirrhotic compared with control livers for Gram-negative *Escherichia coli* (*E coli*) and Gram-positive *Staphylococcus aureus* (*S aureus*) particles, respectively (Figure 5*A* and *B*), and revealed a reduced percentage of macrophages able to take up *E coli* in cirrhosis, and it was similar for *S aureus* (Figure 5*B*). AXL expression on macrophages was unaltered on incubation with bacterial bioparticles (Figure 5*C*). Phagocytosis capacity of bacterial bioparticles of CD68^+^AXL^+^ cells did not differ from CD68^+^AXL^-^ cells (Figure 5*D*).

Because of the accumulation of AXL-expressing cells in the circulation and lymph nodes in cirrhosis, we hypothesized these cells may have migratory properties. Indeed, cells that underwent either transendothelial or reverse migration displayed higher AXL expression than non-migrated cells (Figure 5*E*).

### AXL-Expressing Homeostatic Macrophages Are Reduced in the Gut Mucosa and Peritoneum but Increased in Regional Lymph Nodes in Advanced Cirrhosis

Having described a reduction of AXL expression on liver macrophages and a concurrent accumulation of AXL-expressing circulating monocytes in advanced cirrhosis,^20^ we assessed compartment-specific occurrence and proportion of AXL-expressing macrophages in the gut, the peritoneum, lymph nodes, and the bone marrow.

**Figure 6:**
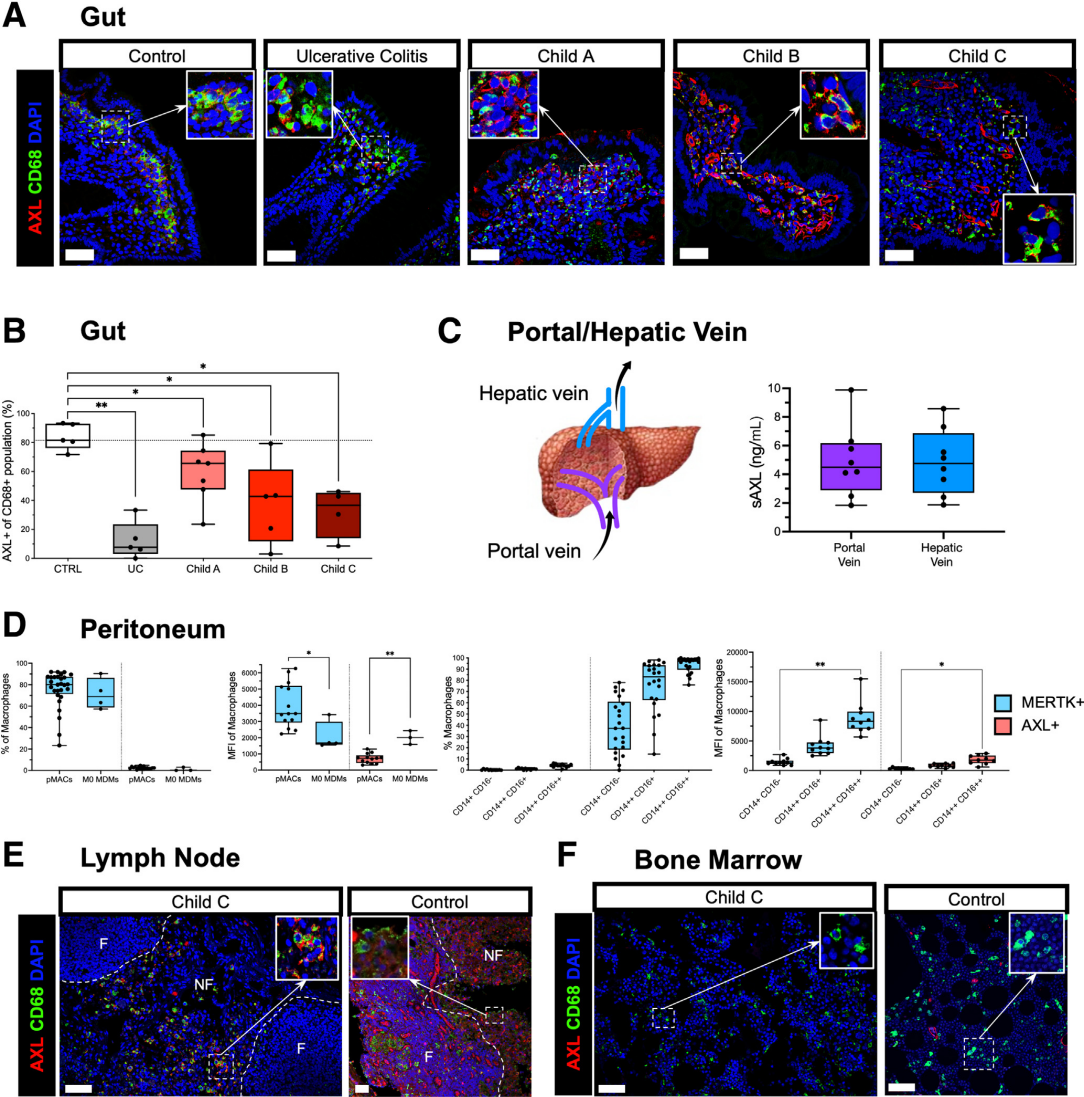
**Prevalence of AXL expression on macrophages in gut, peritoneum, bone marrow, and lymph node.** (*A*) Representative immunofluorescence micrographs from AXL/CD68/DAPI stains of gut biopsies from control group (n = 4), ulcerative colitis (UC) (n = 5), Child-Pugh A (n = 7), Child-Pugh B (n = 5), and Child-Pugh C (n = 4). (*B*) Percentage of AXL^+^ macrophages lining the epithelial barrier relative to total population per HPF. (*C*) Schematic drawing of liver vessels with blood flow indicated and soluble AXL (sAXL) levels (pg/mL) in blood from portal and hepatic veins (n = 8). (*D*) AXL and MERTK expression of peritoneal macrophages from cirrhosis patients (pMACs, n = 23) in classical (CD14^+^CD16^–^, class), intermediate (CD14^++^CD16^+^, inter), and non-classical (CD14^++^CD16^++^, non-class) subsets and monocyte-derived macrophages (M0-MDMs). (*E*) Representative immunofluorescence micrographs from AXL/CD68/DAPI stains of a lymph node from explant patient (Child-Pugh C) and control in follicular (F) and non-follicular (NF) regions. (*F*) Representative micrograph from AXL/CD68/DAPI stains of bone marrow from explant patient (Child-Pugh C) and control. *Box plots* showing median with 10–90 percentile and all points min-max. ∗*P* ≤ .05/∗∗*P* ≤ .01, Mann-Whitney and Kruskal-Wallis test. AF594, *red*; AF488, *green*; AF647, *white*; DAPI, *blue*; scale bar = 50 μm.

On intestinal macrophages from colon biopsies we identified AXL expression on the majority of resident macrophages in healthy gut mucosa, whereas a significant decrease of CD68^+^AXL^+^ macrophages was found in patients with advanced cirrhosis and similarly in conditions of chronic inflammation unrelated to cirrhosis such as in patients with ulcerative colitis (Figure 6*A* and *B*). Plasma levels of soluble AXL from portal venous and hepatic venous blood in patients undergoing transplantation may indicate a slight tendency of higher soluble AXL levels in the hepatic venous blood, albeit no significant difference (Figure 6*C*).

The pMACs isolated from the ascitic fluid of decompensated cirrhotic patients expressed significantly lower levels of AXL but higher levels of MERTK when compared with monocyte-derived M0 macrophages. MERTK expression by pMACs was lowest in the classical and increased in the intermediate and non-classical subsets, whereas AXL expression was low in all subsets but showed slightly elevated levels in the non-classical subset (Figure 6*D*).

From one patient with advanced cirrhosis (Child-Pugh C) who underwent transplantation, we investigated macrophages in mesenteric lymph nodes and observed the presence of CD68^+^AXL^+^ macrophages accumulating in the non-follicular regions. In control lymph nodes, CD68^+^ macrophages did not express AXL; however, AXL was detectable on endothelial cells of vessels (Figure 6*E*). Neither bone marrow from the Child-Pugh C patient nor from control displayed AXL expression (Figure 6*F*).

### Mechanisms Down-Regulating AXL Expression on Liver Macrophages

Next, we aimed to understand the underlying mechanisms as to how AXL expression on liver macrophages was reduced on the evolution of cirrhosis. It has been shown that GAS6, a ligand with high affinity for AXL, down-regulated AXL expression *in vitro*.^23^ Moreover, we previously showed that patients with cirrhosis had elevated plasma GAS6 levels increasing with severity of the disease.^20^ Although the cellular origin of GAS6 in the blood of cirrhotic patients is unknown, a rat model of carbon tetrachloride (CCl_4_)-induced liver injury showed increased expression of GAS6 by aHSCs.^24^ Thus, we hypothesized that GAS6 released by aHSCs in the context of cirrhosis may lead to AXL down-regulation in resident liver macrophages.

**Figure 7:**
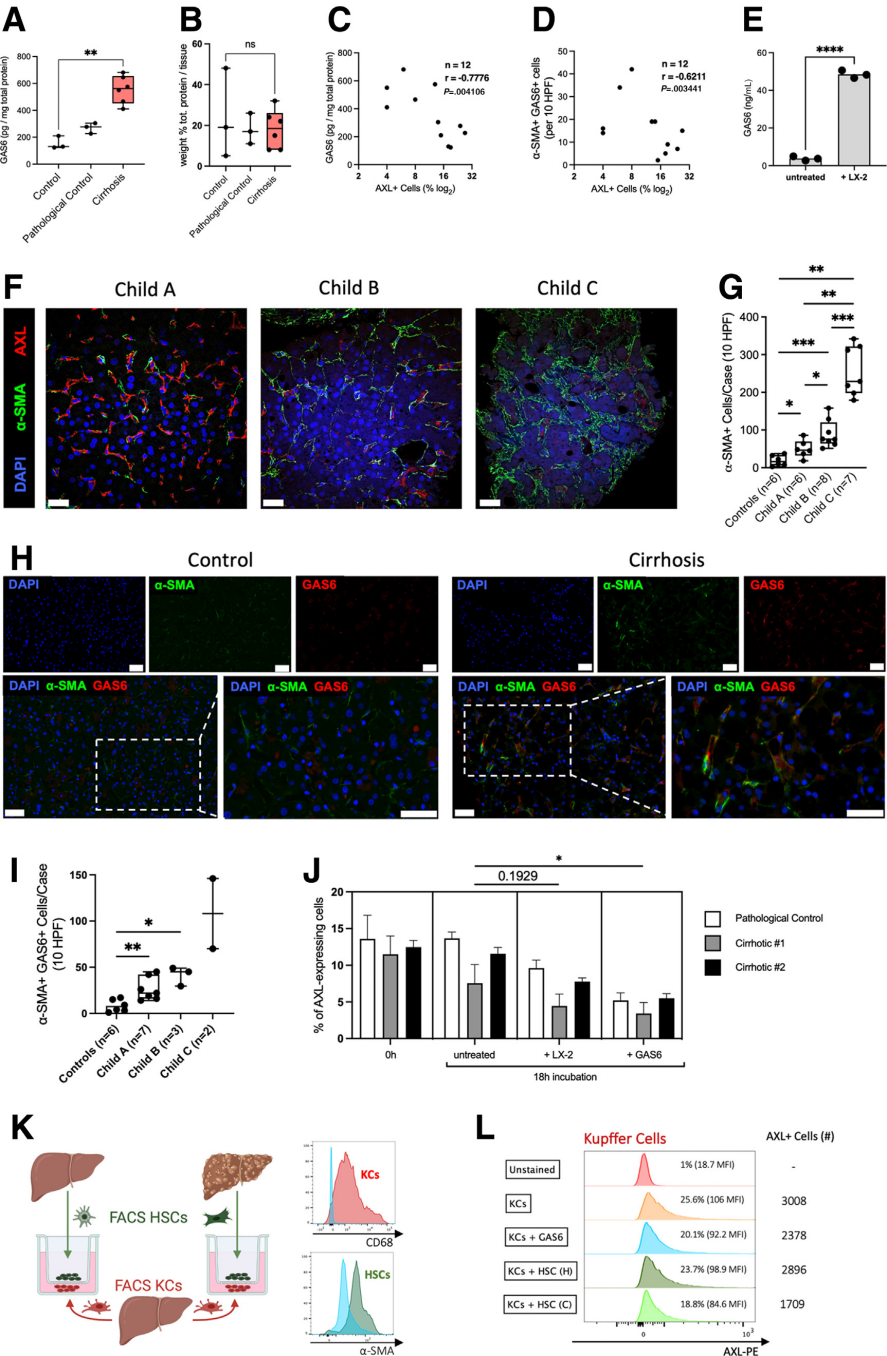
**Mechanism of AXL loss on resident liver macrophages.** (*A*) GAS6 levels in liver tissue lysates and (*B*) protein percentage of liver tissue lysates assessed by bicinchoninic acid assay (BCA) from controls (n = 3), pathologic control (n = 3), and compensated cirrhosis liver resections (n = 6). (*C*) GAS6 levels in liver tissue in correlation with AXL expression on liver macrophages determined by flow cytometry (n = 12, Spearman r correlation). (*D*) α-SMA^+^GAS6^+^ cell numbers (immunofluorescence) in correlation with AXL expression on liver macrophages (flow cytometry) (n = 12, Spearman r correlation). (*E*) GAS6 levels in supernatants after 18 hours of liver macrophage cultures or co-cultures of liver macrophages and LX-2 cells (n = 3). (*F*) Representative immunofluorescence micrographs from α-SMA/AXL/DAPI stains of either liver resections from histologically normal livers (n = 6) or liver biopsies from patients with cirrhosis (Child-Pugh A, n = 6; Child-Pugh B, n = 8; Child-Pugh C, n = 7). AF594, *red*; AF488, *green*; DAPI, *blue*; original magnification, 400×; scale bar = 20 μm. (*G*) Cell count of α-SMA^+^ cells per 10 HPF in healthy controls and patients with cirrhosis. (*H*) Representative immunofluorescence micrographs from α-SMA/GAS6/DAPI stains of liver resections from histologically normal livers (n = 6), as well as liver biopsies and liver resections from patients with cirrhosis (Child-Pugh A, n = 7; Child-Pugh B, n = 3; Child-Pugh C, n = 2). AF647, *red*; AF488, *green*; DAPI, *blue*. Upper panels: original magnification, 400×; scale bar = 50 μm; lower panels: details, scale bar = 50 μm. (*I*) Cell count of α-SMA^+^ GAS6^+^ cells per 10 HPF in healthy controls and patients with cirrhosis. (*J*) AXL expression change in liver macrophages on either co-culture with LX-2 cells or GAS6 treatment (n = 3, technical triplicates); bar plots with standard deviation error, nested *t* tests. (*K*) Experimental setup for co-culture of healthy KCs with either healthy HSCs or cirrhotic HSCs in a transwell system and confirmatory flow cytometry analysis of sorted cells. (*L*) Flow cytometry analysis of either CD68^+^ KCs or α-SMA^+^ HSCs after co-culture experiment depicted in *G* (n = 1). *Box plots* showing median with 10–90 percentile and all points min-max. ∗*P* ≤ .05/∗∗*P* ≤ .01, Mann-Whitney and Kruskal-Wallis test.

Supporting this hypothesis, we measured elevated GAS6 levels in liver tissue lysates from cirrhotic patients compared with controls (Figure 7*A*), and the percentage of total protein was comparable between control and cirrhotic tissue (Figure 7*B*). Both GAS6 levels in liver tissue and α-SMA^+^GAS6^+^ cells inversely correlated with AXL expression on liver macrophages isolated from the same tissue (Figure 7*C* and *D*). Moreover, GAS6 levels were higher in the supernatants of macrophages co-cultured with LX-2 cells, a cell line for aHSCs, compared with liver macrophages cultured *ex vivo* alone (Figure 7*E*). We also performed immunofluorescent staining on liver biopsies and liver resection tissue from patients with cirrhosis and controls showing AXL, α-SMA, and GAS6 expression (Figure 7*F* and *H*). We observed significantly increased numbers of α-SMA^+^ as well as α-SMA^+^GAS6^+^ cells across Child-Pugh stages of cirrhosis, suggesting that GAS6 was produced by aHSCs in the context of disease (Figure 7*G* and *I*). This is in line with recently published data showing that the α-SMA^+^ area significantly increased in patients with nonalcoholic fatty liver disease and advanced fibrosis (F3) or cirrhosis (F4).^25^ For further endorsement, we performed 2 proof-of-principle experiments. Stimulation with GAS6 significantly decreased AXL expression on primary liver macrophages isolated from either 2 patients with cirrhosis or a pathologic control (NCPH) *ex vivo*, and co-culturing with LX-2 cells displayed a trend for reduced AXL expression (Figure 7*J*). To further mimic *in vivo* settings, we co-cultured sorted liver macrophages from a histologically normal liver with sorted HSCs from either a cirrhotic liver or a histologically normal liver. This revealed lower AXL expression and numerically decreased AXL^+^ KCs in cirrhotic conditions and on GAS6 stimulation alone (Figure 7*K* and *L*).

### Axl-Expressing Macrophages Are Reduced in a Murine CCl_4_ Model of Fibrosis

**Figure 8:**
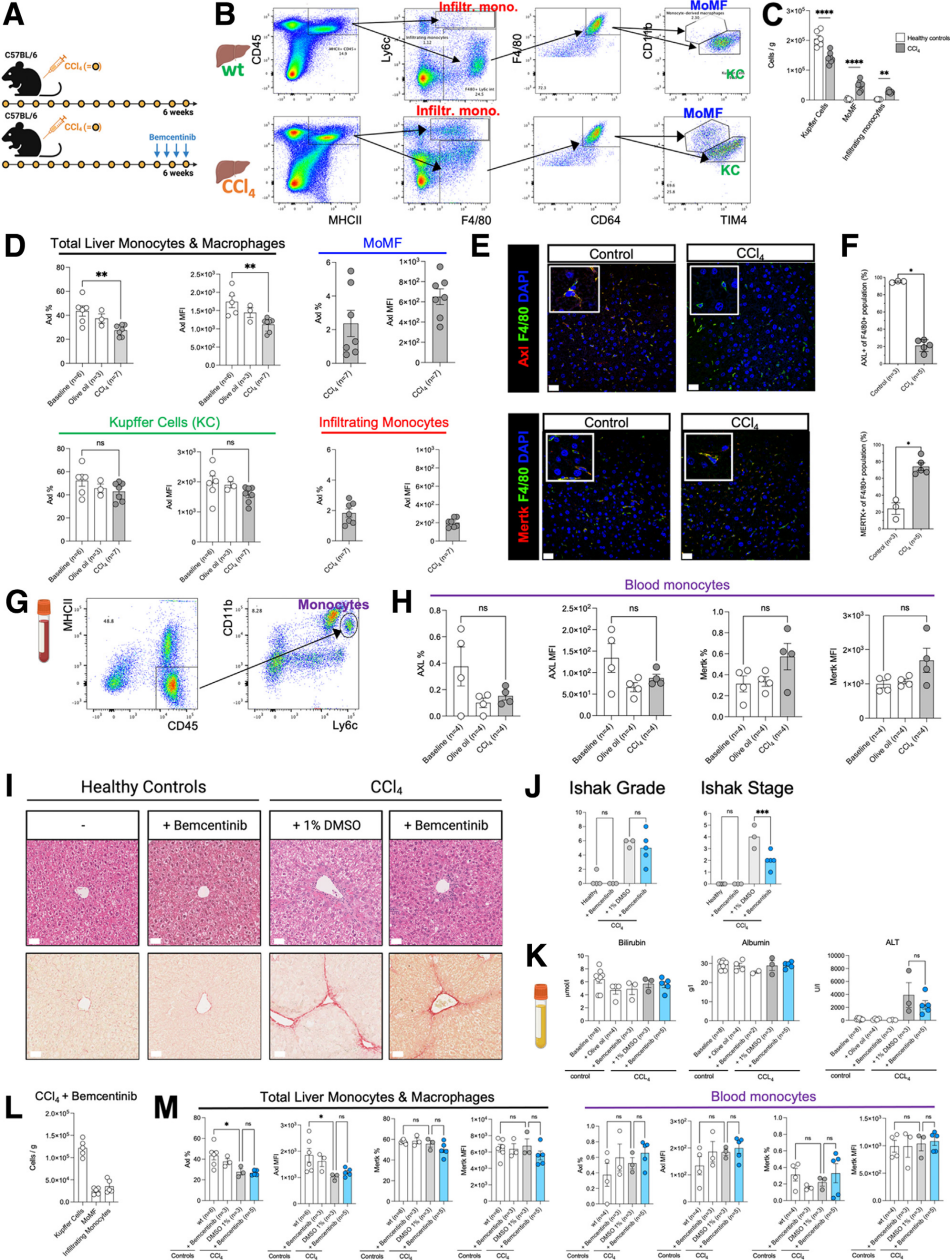
**Efficacy and safety of Axl inhibition in a carbon tetrachloride-induced liver fibrosis model.** (*A*) Overview of carbon tetrachloride (CCl_4_) model involving biweekly administration of CCl_4_ (intraperitoneal 0.4 mL/kg) for 6 weeks and therapeutic immunomodulation with bemcentinib for 1 week every other day (intraperitoneal 100 mg/kg). (*B*) Flow cytometry gating strategies for liver cell subsets including infiltrating monocytes, monocyte-derived macrophages (MoMF), and Kupffer cells (KCs) in a wild-type (wt) and CCl_4_ mouse model. (*C*) Monocyte/macrophage cell numbers per gram of liver tissue in healthy controls and CCl_4_ model. (*D*) Flow cytometry analysis of Axl expression levels displayed as either % or median fluorescence intensity (MFI) on liver cell subsets identified in *B*. (*E*) Representative micrographs from either Axl/F4/80/DAPI stains or Mertk/F4/80/DAPI stains of mouse livers from healthy controls and CCl_4_-treated animals. (*F*) Percentage of either Axl-expressing macrophages or Mertk-expressing macrophages relative to total population (mean per mouse). (*G*) Flow cytometry gating strategy for blood monocytes. (*H*) Flow cytometry analysis of either Axl or Mertk expression levels displayed as % or MFI on blood monocytes identified in G. (*I*) Representative images of hematoxylin-eosin stainings (above) and Sirius Red stainings (below) of FFPE liver sections from either healthy controls or CCl_4_-treated mice with or without bemcentinib treatment. (*J*) Liver necroinflammation score (Ishak grade) and liver fibrosis score (Ishak stage) based on histopathology analysis of H&E and Sirius Red stainings. (*K*) Plasma levels of bilirubin, albumin, and alanine aminotransferase (ALT) assessed with a clinical chemistry analyzer. (*L*) Cells per gram of liver tissue from CCl_4_-treated mice after administration of bemcentinib. (*M*) Axl and Mertk (% and MFI values) on either liver monocytes/macrophages or blood monocytes assessed with flow cytometry analysis. ns = not significant/∗*P* < .05/∗∗*P* < .01, unpaired *t* tests and one-way ordinary analysis of variance. AF594, *red*; AF488, *green*; DAPI, *blue*; scale bar = 50 μm.

Recurrent CCl_4_ treatment is used as a murine model of liver fibrosis.^26^ Thus, we aimed to assess Axl expression levels in liver monocytes and macrophages in a CCl_4_ model using wild-type mice. Subsequently, we assessed the efficacy and safety of Axl inhibition *in vivo* by administering bemcentinib (a small molecule inhibitor of Axl) in CCl_4_-treated mice (Figure 8*A*).

First, to investigate Axl expression in the liver, we isolated hepatic nonparenchymal cells and performed flow cytometry analysis (Figure 8*B*). Although KCs vastly outnumbered other liver monocyte/macrophage subsets in healthy controls, both infiltrating monocytes and monocyte-derived macrophages (MoMF) expanded in the CCl_4_ model (Figure 8*C*). In line with our findings in human livers, Axl expression levels on total liver monocytes and macrophages were reduced in the CCl_4_ model compared with healthy controls in terms of both percentage and expression values. Interestingly, KCs displayed higher Axl expression compared with both infiltrating monocytes and MoMF (Figure 8*D*). In accordance with our flow cytometry data, immunofluorescence staining showed that more than 90% of the F4/80^+^ resident macrophages in control murine liver tissue expressed Axl, whereas F4/80^+^Axl^+^ macrophages were significantly decreased in livers from CCl_4_-treated mice. Moreover, F4/80^+^Mertk^+^ macrophages increased compared with healthy controls (Figure 8*E* and *F*). Using flow cytometry analysis of blood (Figure 8*G*), we did not observe differences in phenotype of blood monocytes between the CCl_4_ model and controls (Figure 8*H*).

Next, we assessed the potential efficacy and safety of bemcentinib administration in our CCl_4_ model and performed histopathology analyses on both hematoxylin-eosin and Sirius Red stainings of formalin-fixed and paraffin-embedded (FFPE) liver sections from bemcentinib-treated CCl_4_ mice (Figure 8*I*). Although Ishak grade, a scoring system for liver necroinflammation, indicated no change after bemcentinib administration compared with CCl_4_ controls, Ishak stage, a scoring system for liver fibrosis, was significantly reduced after bemcentinib administration (Figure 8*J*), which is in accordance with findings in murine nonalcoholic steatohepatitis.^27^ Importantly, plasma levels of bilirubin and albumin as well as plasma levels of alanine transaminase were unvaried after administration of bemcentinib in the CCl_4_ model, underlining its safety relating to the liver for therapeutic use (Figure 8*K*). Bemcentinib administration had no effect on frequency of liver monocyte/macrophage subsets compared with CCl_4_ controls (Figure 8*L*). Moreover, Axl and Mertk on both liver monocytes/macrophages and blood monocytes were unvaried after therapeutic immunomodulation with bemcentinib (Figure 8*M*).

## Discussion

Previously we identified the emergence of circulating immune-modulatory monocytes expressing AXL in parallel with disease progression of cirrhosis that dampened immune responses to microbial challenge.^20^^,^^27^ The role of AXL expression on tissue macrophages in the liver and associated compartments such as gut and peritoneum in the context of immune-homeostasis regulation in health and cirrhosis as a multisystem disorder remained unknown.

In this study we newly identified the ubiquitous expression of AXL on resident liver macrophages in healthy livers, whereas endothelial cells, HSCs, and hepatocytes did not express AXL. In cirrhosis, AXL expression on KCs was significantly reduced in association with disease severity and infectious complications. In patients with cirrhosis, we also observed a reduced expression of AXL on gut and peritoneal macrophages, whereas AXL expression was elevated on macrophages in mesenteric lymph nodes. Because of these distinct expression patterns, we hypothesize that regulatory mechanisms of AXL expression are presumably disease- and compartment-specific. As a mechanism, we showed previously that AXL expression on monocytes was enhanced in response to microbial products and efferocytosis,^20^ which may explain their abundance in the circulation in the context of cirrhosis. Data from this study hint at a mechanism involving GAS6, produced by aHSCs in the cirrhotic liver, potentially down-regulating AXL on human liver macrophages.

Phenotypically, in line with our previous findings on monocytes,^20^ AXL-expressing macrophages were characterized CD16^high^HLA-DR^high^, indicating a mature macrophage. In addition, higher expression levels of the mannose receptor CD206 were detected, which is known to identify tissue macrophages and for its essential function in phagocytosis and immune homeostasis by scavenging adverse mannoglycoproteins.^28^

Functionally, phagocytosis capacity for pathogens was unchanged between AXL^+^ and AXL^–^ macrophages, similarly to our recently published data, where we observed AXL-expressing circulating monocytes characterized by preserved phagocytosis of pathogens and enhanced efferocytosis capacity.^20^ Nevertheless, liver macrophages in cirrhosis patients presented reduced phagocytosis capacity of Gram-negative bacteria unrelated to AXL. Because of the heterogeneity, there may be other macrophage subsets responsible for this observation. Also in the literature, TAM receptors have been described to be dispensable in the phagocytosis and killing of bacteria.^29^

In cirrhosis, we observed a significant decrease in AXL-expressing resident liver macrophages in parallel with disease severity. AXL levels on resident liver macrophages were similarly decreased in patients with NCPH, suggesting potential involvement of mechanisms eliciting portal hypertension. In line with these findings on protein level, within open available databases of a recently published study using unbiased large-scale techniques, transcriptomes of single liver cells revealed higher AXL expression on KCs of uninjured controls compared with cirrhotic human livers,^26^ which is similar to the results from a dataset of single-cell transcriptomes of KCs from a murine nonalcoholic fatty liver disease/nonalcoholic steatohepatitis model.^30^ Sequential assessment of individual patients, in whom the clinical cirrhosis stage progressed or regressed, demonstrated the relevance of our trans-sectional data. We revealed in exemplary cases that AXL-expressing macrophages undergo a dynamic process depending on disease evolution. Because it is rarely clinically indicated to take a biopsy in patients with biopsy-proven cirrhosis who clinically improved while cirrhosis persisted, trans-sectional biopsy data for cirrhosis regression were highly limited. Hence, further studies are necessary to investigate the dynamics of AXL expression on KCs during cirrhosis evolution. We also studied AXL on KCs of patients with advanced cirrhosis who underwent transplantation and observed an increase of AXL expression after transplantation. Even though liver transplantation does not represent regression of the disease, interestingly it has been shown that recipient leukocytes rapidly repopulated the transplanted liver and were reprogrammed toward CD68^+^/CD206^+^ liver macrophages, whereas only a small residual population of donor KCs persisted.^31^^,^^32^

In line with previous data from our group,^18^ we observed few MERTK-expressing macrophages in healthy liver tissue. Instead, their accumulation occurred in cirrhotic livers in relation to disease severity and inversely to the reduction of AXL expression. Moreover, AXL and MERTK did not co-localize on human macrophages. We therefore hypothesize a distinct regulation of AXL and MERTK on liver macrophages as suggested in previous studies on bone marrow–derived macrophages and DCs: MERTK was expressed in response to tolerogenic and AXL in response to inflammatory stimuli.^11^ Under homeostatic conditions in mice in turn, Axl and Mertk co-expression has previously been observed on murine KCs^17^ and on murine lung airway macrophages.^12^

KCs represent ∼20% of all liver cells and ∼80% of body’s tissue macrophages.^33^ They are pivotal sinusoidal cells maintaining hepatic immune homeostasis and tolerance due to their ability to clear pathogens and their products derived from the intestine.^34^ Balmer et al^1^ proposed that the liver may act as a vascular firewall of the systemic circulation facilitating phagocytosis of commensal organisms derived from the intestine. TAM receptors have been generally described to maintain homeostasis and promote phagocytic removal of apoptotic cells (efferocytosis) during resolution phases of inflammation.^9^^,^^10^ In the liver, TAM receptor triple knockout mice developed spontaneous chronic hepatic inflammation.^35^ DCs of triple knockout mice showed a hyper-responsiveness to endotoxins and impaired efferocytosis.^9^ In acute liver injury models, more severe parenchymal damage was observed in Axl^-/-^ compared with Mertk^-/-^ mice; nonetheless, Mertk was required for efferocytosis of apoptotic cells.^17^ Besides being involved in immune homeostasis, MERTK and AXL were also associated with deleterious processes in some liver diseases.^36^ In mouse models of nonalcoholic steatohepatitis^27^ and fibrosis,^17^^,^^37^ Axl signaling was associated with increased inflammation^27^ and fibrogenesis^17^^,^^27^^,^^37^, whereas Mertk signaling was involved in protection of hepatocytes against lipotoxicity.^27^ The respective effects of AXL and MERTK thus depend on expressing cell types and compartments, disease stage, and the degree of expression. Axl-expressing macrophages have been particularly implicated in immunologic barrier functions in various compartments. Axl was expressed under homeostatic conditions on murine airway but not interstitial lung macrophages. Axl-expressing airway macrophages expanded after influenza infection, thereby preventing excessive tissue inflammation through efferocytosis.^12^ In the central nervous system, Axl was highly expressed on microglia in the context of neurodegeneration^13^ and autoimmune encephalomyelitis^14^ and associated with efferocytosis of myelin debris. In the joint, murine tissue-resident synovial lining CX_3_CR1^+^ macrophages expressed a high level of Axl in their transcriptional profiling and formed an internal immunologic barrier as a protective shield for intra-articular structures.^15^ Of note, we did not detect AXL expression on bone marrow macrophages, highlighting their suggested role in microbial defense in physiological conditions.

In cirrhosis with emerging portal hypertension, the load of translocated bacteria and bacterial products from the gut to the systemic circulation is enhanced, which is termed as pathologic bacterial translocation.^38^^,^^39^ Here, we question whether decreased AXL expression on macrophages in the cirrhotic liver may relate to reduced immune tolerance and barrier protection in liver tissue and enhanced liver inflammation in the context of elevated load of translocated microbes and pathogen-associated molecular patterns in advanced cirrhosis. On the other hand, the concurrent accumulation of AXL-expressing monocytes in mesenteric lymph nodes and in the circulation^20^ may possibly favor a state of systemic tolerance, i.e.e, immuneparesis in cirrhosis patients. The following arguments may support this hypothesis. AXL expression on innate immune cells has been allocated to barrier compartments as described above. Moreover, the function of AXL-expressing cells (including enhanced efferocytosis, preserved phagocytosis, reduced cytokine production, and reduced T-cell activation as reported previously^20^) reflects an innate immune state at the interface of exposure to potential pathogens and tolerance to commensal microbes. Finally, there are the mechanisms up-regulating AXL expression upon efferocytosis, phagocytosis, and stimulation with microbial products (TLR agonists).^20^

We also confirmed that murine resident liver macrophages showed reduced Axl expression on CCl_4_-induced fibrosis. However, it must be considered that although CCl_4_ induces fibrosis and models using CCl_4_ + TLR ligands such as lipopolysaccharide may mimic inflammatory events occurring on top of fibrosis, murine models reflecting the advanced stage of cirrhosis as a multisystemic disease do not exist. Reduction in Axl in CCl_4_-induced fibrosis was mainly caused by reduction of KCs and substitution by Axl^low^ MoMF and Axl^low^ infiltrating monocytes. As expected, Axl on circulating mouse monocytes in this stage of fibrosis did not differ from controls, considering our previous data^20^ describing an expansion of AXL-expressing circulating monocytes in patients with cirrhosis. Our finding partially corroborates previously published murine data (C57/BL6 background), where Axl was expressed on hepatic KCs but also on endothelial cells.^17^ In their CCl_4_ model, hepatic Axl up-regulation was observed, whereby Axl-expressing cell types have been detailed by immunofluorescence without quantification only, whereas we used immunofluorescence and flow cytometry to quantify AXL on KCs.^17^

The idea of using a selective Axl inhibitor (bemcentinib) in a CCl_4_ model was to evaluate the hepatic and systemic effects of AXL inhibition in the context of fibrosis. We recently described the occurrence of AXL-expressing circulating monocytes in cirrhosis characterized by impaired inflammatory responses, thereby favoring immuneparesis, which were reversed by bemcentinib *ex vivo*.^20^ At certain stages of cirrhosis, the effect of AXL inhibition might be desirable in the systemic circulation but not necessarily in the liver, gut, and presumably other tissues because liver and gut macrophages revealed reduced AXL expression in advanced cirrhosis. Nevertheless, in our CCl_4_ model, bemcentinib did not enhance hepatic inflammation and also reduced fibrosis, as previously shown by other groups through its inactivating effect on HSCs^37^ and prevention of liver fibrosis/inflammation in early nonalcoholic steatohepatitis.^27^ This may imply first evidence that AXL inhibitors may be safely used and further evaluated in patients with cirrhosis. How AXL inhibition might eventually find therapeutic application in humans with regard to its particular compartmental expression profiles in health and disease conditions needs to be carefully dissected in future murine *in vivo* studies to investigate distinct target and off-target effects.

In extrahepatic sites, we discovered AXL expression on resident macrophages in healthy gut tissue, whereas it was lower in patients with cirrhosis and ulcerative colitis, supporting our hypothesis of AXL being a crucial player in maintaining immune homeostasis by providing defense against microbes not only in the liver. In agreement with this, high expression of genes associated with phagocytosis, such as *Mertk, Gas6,* and *Axl,* has been described in murine intestinal macrophages.^40^ On the other hand, activated intestinal macrophages in patients with cirrhosis have been associated with an increased intestinal permeability enhancing the translocation of pathogens by releasing interleukin-6 and nitric oxide.^41^ In summary, in chronic inflammatory conditions, the invasion of pathogens from the intestine might be favored by reduced AXL expression on macrophages, thereby promoting disease progression and inflammation.

Similarly, we observed low AXL but increased MERTK expression on peritoneal macrophages of decompensated cirrhosis patients with ascites. Generally, little is known about healthy human pMACs because of limited sample availability. Murine pMACs have been shown to strongly express Mertk^12^^,^^42^ (and no Axl^12^), which mediated apoptotic cell engulfment.^42^ It remains unknown whether pMACs express AXL under physiological or disease conditions other than cirrhosis.

Although translocated bacterial products as well as pathogen and apoptotic cell uptake lead to an increase of AXL expression on circulating monocytes in patients with advanced cirrhosis,^20^ it remained unknown which mechanisms underlie the down-regulation on macrophages. Because of the enhanced migratory potential of AXL-expressing monocytic cells and their enhancement in other compartments in the condition of advanced cirrhosis, it is conceivable that AXL-expressing cells may egress the liver and migrate towards regional lymph nodes and/or the systemic circulation. Our data do not support the assumption that in cirrhosis AXL^+^ macrophages may be transformed towards MERTK^+^ macrophages, because we did not see that loss of AXL would lead to an up-regulation of MERTK on primary human macrophages *ex vivo* (data not shown). Moreover, a transformation of AXL- into MERTK-expressing cells has to our knowledge not yet been described in the literature.^9^, ^10^, ^11^ Rather, shown by proof-of-principle experiments here, we think that enhanced GAS6 production by aHSCs in the cirrhotic liver may down-regulate AXL expression on primary liver macrophages. As a limiting factor, however, it must be considered that the relevant experiments could only be conducted with small sample sizes because of the requirement of highly limited resources such as primary HSCs and KCs from patients with and without cirrhosis, respectively, undergoing liver resections. GAS6 has been shown to down-regulate AXL in prostate cancer cells,^23^ GAS6 production by aHSCs has previously been described in the context of fibrosis,^24^ and its deficiency reduced fibrosis in mice.^37^ GAS6 is also known to be expressed in chronic inflammatory conditions,^43^ and GAS6 deficiency has been shown to prevent liver inflammation and fibrosis, potentially by restoring AXL on KCs.^44^ Because of the dynamic changes of AXL expression demonstrated by our longitudinal data, inactivation, senescence, and apoptosis of aHSCs^45^ might lower levels of GAS6, thereby permitting AXL re-expression on macrophages. In summary, the reduction of AXL expression on liver macrophages on the evolution of cirrhosis presumably involves different mechanisms, potentially including GAS6-mediated down-regulation of AXL and migration of AXL-expressing monocytic cells to extrahepatic compartments. To what extent these mechanisms interact in reducing AXL expression requires further investigation such as genetic manipulation of these pathways in animal models or the use of high-dimensional analyses. Moreover, we did not investigate the mechanism underlying AXL down-regulation on intestinal macrophages. The role of AXL expression on gut macrophages in intestinal inflammation and cirrhosis is subject of ongoing studies.

In conclusion, we identified AXL as a constitutively expressed tyrosine kinase on resident liver macrophages known to maintain liver tolerance. AXL expression on macrophages was lost in the process of fibrosis progression and portal hypertension in cirrhosis and implicated infectious complications. The mechanism may involve GAS6 production by aHSCs, a process deserving future evaluation in relation to potential immune-modulatory and anti-fibrotic therapies involving the GAS6-AXL axis.

## Materials and Methods

### Patients and Sampling

A cohort of 33 patients with cirrhosis was identified at the University Hospital Basel and the Cantonal Hospital St. Gallen, Switzerland between January 2016 and April 2021 and 29 patients with cirrhosis and ascites at King’s College and St. Mary’s Hospitals, London, United Kingdom, between January 2014 and August 2018. Patients were recruited and categorized according to Child-Pugh scores (Child-Pugh A [biopsy n = 8, resection n = 9], B [biopsy n = 7, resection n = 2], and C [biopsy n = 7]). We further included control subjects such as healthy (biopsy n = 4), patients with NCPH due to nodular regenerative hyperplasia (biopsy n = 4) or chronic liver disease without cirrhosis (biopsy n = 8). All subjects provided written informed consent. Histologically normal non-lesional, cirrhotic, and NCPH liver tissues for immunohistochemistry were obtained from liver biopsy or resection. Histologically normal non-lesional and cirrhotic liver tissues for macrophage isolation were obtained from patients undergoing surgical liver resection for solitary colorectal metastasis and echinococcosis (control n = 14) or hepatocellular carcinoma (cirrhosis n = 11). Exclusion criteria were age younger than 18 years, immunosuppressive therapy, and human immunodeficiency virus infection. The study had been approved by the local ethics committees (EKSG 15/074/EKNZ 2015-308, BASEC-ID 2019-00816, BASEC-ID 2019-02118; UK: 12/LO/0167) and recorded in the clinical trial register ClinicalTrials.gov (identifier: NCT04116242) and Swiss National Clinical Trials Portal (SNCTP000003482).

### Clinical, Hematologic, and Biochemical Parameters

Full blood count, liver and renal function tests, and clinical variables were entered prospectively into a database. The following disease severity scores were calculated, Child–Pugh and MELD, and infections were documented.

### Fluorescent Immunohistochemistry and Confocal Microscopy

Tissue sections, fluorescent immunohistochemistry staining, and confocal microscopy were undertaken as previously described.^18^ Tissue samples were FFPE. The 4-μm serial sections were cut and placed on slides. Single and multiplexed immunofluorescence on FFPE tissue was performed on serial sections to assess CD68, AXL, MERTK, MAC387, CD31, CD34, vWF, α-SMA, GAS6, collagen I, and F4/80 expression.

For CD68 and AXL, sections were incubated for 18 hours at 4°C with a goat anti-human AXL antibody (R&D Systems, catalogue number AF154, lot number DMG0618111, dilution 1:200) in 1× phosphate-buffered saline (PBS), followed by 1-hour incubation at room temperature with an Alexa Fluor 594 donkey anti-goat immunoglobulin (Ig) G (H+L) antibody (Invitrogen, catalogue number A11058, lot number 1842799, dilution 1:400) in 1× PBS. Slides were then incubated again for 18 hours at 4°C with a monoclonal mouse anti-human CD68 antibody (Agilent, clone PG-M1, catalogue number M086, lot number 20029531, dilution 1:100), followed by 1-hour incubation at room temperature with an Alexa Fluor 488 donkey anti-mouse Ig G (H+L) antibody (Jackson Immunoresearch, catalogue number 715-545-150, dilution1:400) in 1× PBS. Slides were then counterstained with DAPI and mounted using fluorescence mounting medium (Dako, catalogue number S3023).

For AXL, MERTK, and collagen I, sections were incubated for 18 hours at 4°C with a goat anti-human AXL antibody (R&D Systems, catalogue number AF154, lot number DMG0618111, dilution 1:250) in 1× PBS, followed by 1-hour incubation at room temperature with Alexa Fluor 594 donkey anti-goat Ig G (H+L) antibody (Invitrogen, catalogue number A11058, lot number 1842799, dilution 1:400) in 1× PBS. This was followed by another incubation for 18 hours at 4°C with a monoclonal rabbit anti-human MERTK antibody (Abcam, clone Y323, catalogue number ab52968, lot number 1829926, dilution 1:200) and 1-hour incubation at room temperature with fluorescein goat anti-rabbit Ig G (H+L) antibody (Invitrogen, catalogue number F2765, lot number 1829926, dilution1:400) in 1× PBS. A third overnight incubation with a monoclonal mouse anti-human collagen I antibody (Abcam, catalogue number ab88147, clone 3G3, dilution 1:300) labeled with Alexa Fluor 647 using a labeling kit from Invitrogen (catalogue number A20186) was followed by counterstaining with DAPI.

For CD68, AXL, and MAC387, sections were incubated for 18 hours at 4°C with goat anti-human AXL antibody (R&D Systems, catalogue number AF154, lot number DMG0618111, dilution 1:200) in 1× PBS, followed by 1-hour incubation at room temperature with Alexa Fluor 594 donkey anti-goat Ig G (H+L) antibody (Invitrogen, catalogue number A11058, lot number 1842799, dilution 1:400) in 1× PBS. Slides were then incubated again for 18 hours at 4°C with monoclonal mouse anti-human CD68 antibody (Agilent, clone PG-M1, catalogue number M086, lot number 20029531, dilution 1:100), followed by 1-hour incubation at room temperature with Alexa Fluor 488 donkey anti-mouse Ig G (H+L) antibody (Jackson Immunoresearch, catalogue number 715-545-150, dilution1:400) in 1× PBS. A third overnight incubation with monoclonal mouse anti-human S100A9 + calprotectin (S100A8/A9 complex) antibody (Abcam, catalogue number ab22506, lot number GR309896-4, clone MAC387, dilution 1:300) labeled with Alexa Fluor 647 using a labeling kit from Invitrogen (catalogue number A20186) was followed by counterstaining with DAPI.

For AXL and CD31/CD34/vWF, sections were incubated for 18 hours at 4°C with a cocktail of primary antibodies: goat anti-human AXL antibody (R&D Systems, catalogue number AF154, lot number DMG0618111, dilution 1:200), monoclonal mouse anti-human CD31 (Agilent, catalogue number M0823, clone JC70A, lot number 20049471, dilution 1:50), monoclonal mouse anti-human CD34 Class II (Agilent, catalogue number M7165, clone QBEnd 10, lot number 20055214, dilution 1:50), and mouse anti-human vWF (Invitrogen, catalogue number MA5-14029, clone F8/86, lot number TF2582851, dilution 1:20) in 1× PBS, followed by 1-hour incubation at room temperature with a cocktail of secondary antibodies: Alexa Fluor 594 donkey anti-goat IgG (H+L) (Invitrogen, catalogue number A11058, lot number 1842799, dilution 1:400) and Alexa Fluor 488 donkey anti-mouse IgG (H+L) antibody (Jackson Immunoresearch, catalogue number 715-545-150, dilution 1:400) in 1× PBS. Slides were counterstained with DAPI.

For AXL and α-SMA, sections were incubated for 18 hours at 4°C with goat anti-human AXL antibody (R&D Systems, catalogue number AF154, lot number DMG0618111, dilution 1:200) in 1× PBS, followed by 1-hour incubation at room temperature with Alexa Fluor 594 donkey anti-goat IgG (H+L) antibody (Invitrogen, catalogue number A11058, lot number 1842799, dilution 1:400) in 1× PBS. Slides were then incubated again for 18 hours at 4°C with monoclonal mouse anti-human smooth muscle actin antibody (Agilent, catalogue number M0851, clone 1A4, lot number 20049711, dilution 1:150), followed by 1-hour incubation at room temperature with a fluorescein goat anti-mouse IgG (H+L) antibody (Invitrogen, catalogue number F2761, lot number 1820001, dilution 1:400) in 1× PBS. Slides were counterstained with DAPI.

For AXL and F4/80, sections were incubated for 18 hours at 4°C with a cocktail of antibodies: goat anti-mouse AXL antibody (R&D Systems, catalogue number AF854, lot number CTC0218071, dilution 1:20) and monoclonal Alexa Fluor 647 rat anti-mouse F4/80 antibody (BioLegend, catalogue number 123122, clone BM8, dilution 1:50) in 1× PBS. Sections were then incubated for 1 hour at room temperature with Alexa Fluor 594 donkey anti-goat IgG (H+L) antibody (Invitrogen, catalogue number A11058, lot number 1842799, dilution 1:400) in 1× PBS. Slides were counterstained with DAPI.

For Mertk and F4/80, sections were incubated for 18 hours at 4°C with a cocktail of antibodies: goat anti-mouse Mertk antibody (R&D Systems, catalogue number AF591, lot number DGS0517091, dilution 1:20) and monoclonal Alexa Fluor 647 rat anti-mouse F4/80 antibody (BioLegend, catalogue number 123122, clone BM8, dilution 1:50) in 1× PBS. Sections were then incubated for 1 hour at room temperature with Alexa Fluor 594 donkey anti-goat IgG (H+L) antibody (Invitrogen, catalogue number A11058, lot number 1842799, dilution 1:400) in 1× PBS. Slides were counterstained with DAPI.

### Image Acquisition and Analysis

All micrographs for fluorescent images were acquired using an LSM980 confocal microscope with Airyscan 2 (Zeiss, DE). For the quantitative morphometry, 10 HPF (400× magnification) were acquired for each slide, and the number of positive cells per HPF was assessed for the following combinations of markers: CD68^+^, F4/80^+^, CD68^+^AXL^+^, AXL^+^MERTK^+^, MAC387^+^AXL^+^, CD31^+^CD34^+^vWF^+^AXL^+^, α-SMA^+^AXL^+^, F4/80^+^Axl^+^, and F4/80^+^Mertk^+^.

### Human Macrophage Isolation

Small resection samples (<5 cm × 5 cm × 1 cm) were mechanically disrupted using a filter mesh,^46^ whereas large resection samples were processed using a Stomacher 400 circulator (Seward).^19^

### Flow Cytometry-Based Phenotyping of Hepatic Macrophages

Phenotyping of hepatic macrophages isolated from liver resection specimen was undertaken using flow cytometry (LSRFortessa Cell Analyzer, BD Biosciences) as previously described.^18^ Flow cytometry antibodies were purchased from the indicated companies below. Flow cytometry data were analyzed using FlowJo software (V.10.7.1, Becton Dickinson & Company) including the clustering and visualization technique of FlowSOM.^47^

Antibody information is as follows: BD Biosciences: CD14-PE-Cy7 (557742), CD16-BV650 (563692), CD64-FITC (555527), CD163-PE (556018), CD11b-PE (555388), isotype controls Mouse IgG1 κ BV650 (563231), Mouse IgG2a κ Pe-Cy7 (557907); R&D Systems: AXL-AF488 (FAB154G), MERTK-APC (FAB8912A), IFNAR-PE (FAB245P), S100A8/A9-AF488 (IC9337G); BioLegend: HLA-DR-PerCP-Cy5.5 (307630), TLR4-APC (312816), CD206-BV421 (321126), CCR2-BV510 (357218), CCR5-BV421 (359118), CCR7-BV421 (353208), CX3CR1-BV510 (341621), CXCR4-BV510 (306536), CD68-BV785 (333826), CD3-APC-Fire750 (300470), CD19-APC-Fire750 (302258), CD56-APC-Fire750 (362554), Mouse IgG2b κ BV785 (400355), Mouse IgG2a κ PerCP-Cy5.5 (400252), Mouse IgG1 κ APC-Fire750 (400196); and eBioscience: CD32-FITC (11-0329-42), Fixable Viability Dye eFluor 455UV (65-0868-14) for cell viability, and FOXP3/Transcription Factor Staining Buffer Set (00-5523-00) for intracellular staining.

### Flow Cytometry–Based Phagocytosis Assay in Hepatic Macrophages

Cells were incubated with pHrodo *E coli* (P35361) or *S aureus* (A10010) Red BioParticles (Phagocytosis Kit for Flow Cytometry from Invitrogen/Thermo Fisher Scientific) according to the manufacturer’s protocol. At least 100,000 cells in 100 μL FACS Buffer supplemented with 10% human AB serum were incubated with 5 μL of BioParticles for 30 minutes at 37°C. Cells were fixed and stained with antibodies.

### Flow Cytometry–Based Phenotyping of Peritoneal Macrophages

The pMACs from ascites were obtained from ascitic tap fluid by centrifugation. The pMACs were stained after isolation by centrifugation (500*g*, 5minutes) and stained for AXL, MERTK, CD14, CD16, and HLA-DR for flow cytometry as previously described.^18^

### In Vitro Co-Culture Experiments of Hepatic Macrophages With LX-2 Cells, Primary Hepatic Stellate Cells, or Treatment With GAS6

Hepatic macrophages were isolated from liver resection specimens, plated for 18 hours in RPMI 1640 medium (10% fetal bovine serum, 1% Pen/Strep), and either co-cultured with LX-2 cells (an aHSCs cell line^48^; gift of Prof Dr Scott Friedman, Mount Sinai School of Medicine) at a ratio of 1:1 in a transwell system (cellQART, cat. #9320402) or treated with GAS6 (500 ng/mL) (885-GSB R&D Systems). In addition, hepatic macrophages were isolated with fluorescence-activated cell sorting from liver resection specimens and co-cultured in a transwell system with either sorted aHSCs from histologically normal liver tissue or sorted aHSCs from cirrhotic tissue. Subsequently, cells were harvested and phenotypically characterized by surface and intracellular marker expression levels via flow cytometry.

### Protein Extraction From Snap-Frozen Liver Tissues

Snap-frozen tissue samples from resection specimens were lysed using metallic beads and radioimmunoprecipitation lysis buffer system (ChemCruz, cat. Sc-24948). Samples were lysed in the tissue lyser (Qiagen) for 5 minutes at 25*g*. Supernatants underwent 3 thawing/freezing cycles before centrifugation (20,800*g*, 20 minutes).

### Total Protein, GAS6, Soluble AXL Level Assessment

Total protein levels in liver tissue lysates from resection specimens were assessed with a bicinchoninic acid assay. Briefly, colorimetric detection of bicinchoninic acid-Cu1+ complexes was detected at 562 nm using a microplate reader (BioTek Synergy H1), indicating total protein concentrations. GAS6 levels in liver tissue lysates from resection specimens and in supernatant from *in vitro* experiments were assessed using a GAS6 ELISA kit (BMS2291, ThermoFisher Scientific). Soluble AXL levels in serum samples were measured using a sAXL ELISA kit (ab99976, Abcam).

### Migration Assay

Transendothelial migration of monocytes across stimulated endothelium was assessed using an established^49^ *in vitro* migration assay and performed according to a previously published protocol.^18^

### Carbon Tetrachloride–Induced Liver Fibrosis Model

Wild-type C57BL/6 8- to 10-week-old male mice (Charles River, UK) were administered 0.4 mL/kg CCl_4_ (Sigma-Aldrich), diluted in olive oil (Sigma-Aldrich) at a 1:3 ratio. Mice were injected intraperitoneally twice a week for total of 6 weeks (n = 13 intraperitoneal CCl_4_ injections). In addition, a group of wild-type mice were administered bemcentinib (Selleckchem, cat. #S2841) intraperitoneally at 100 mg/kg dose 4 times over the last week before death. Twenty-four hours after the last CCl_4_ injection, mice were killed, livers were perfused with PBS, and liver tissue was FFPE for fluorescent immunohistochemistry analysis. All animal experimental protocols were approved by Imperial College London in accordance with UK Home Office regulations (PPL numbers: 70/7578 and P8999BD42).

### Flow Cytometry–Based Phenotyping of Murine Liver Monocytes/Macrophages and Blood Monocytes

Mouse hepatic non-parenchymal cells were isolated as previously described,^50^ and liver monocytes and macrophages were phenotypically characterized by flow cytometry. Cells were incubated for 30 minutes at 4°C with a viability dye (Zombie Live Dead, #423101, BioLegend) and the following flow cytometry antibodies: BioLegend: F4/80-BV421 (cat. #123137), CD45-BV650 (cat. #103151), CD11b-BV711 (cat. #101242), CD64-PerCP-Cy5.5 (cat. #139308), Ly6c-PE-Cy7 (cat. #128018); eBioscience: Tim4-AF488 (cat. #53-5866-82), Mertk-AF700 (cat. #56-5751-82), MHCII-APC-eFluor780 (cat. #47-5321), and Axl-PE (R&D cat. #FAB8541P). Blood monocytes were phenotypically characterized with flow cytometry using the same antibodies.

### Statistical Analysis

Data are expressed as the median/interquartile range unless otherwise specified. For data that did not follow a normal distribution, the significance of differences was tested using the Mann–Whitney or Wilcoxon tests; Spearman correlation coefficients were calculated. Graphs were drawn using Prism 9.3.0 (GraphPad, La Jolla, CA).

## References

[1] Balmer M.L., Slack E., Gottardi A de (2014). The liver may act as a firewall mediating mutualism between the host and its gut commensal microbiota. Sci Transl Med.

[2] Bernsmeier C., van der Merwe S., Périanin A. (2020). Innate immune cells in cirrhosis. J Hepatol.

[3] Albillos A., Lario M., Álvarez-Mon M. (2014). Cirrhosis-associated immune dysfunction: distinctive features and clinical relevance. J Hepatol.

[4] Wiest R., Lawson M., Geuking M. (2014). Pathological bacterial translocation in liver cirrhosis. J Hepatol.

[5] Stengel S., Quickert S., Lutz P. (2020). Peritoneal level of CD206 associates with mortality and an inflammatory macrophage phenotype in patients with decompensated cirrhosis and spontaneous bacterial peritonitis. Gastroenterology.

[6] Arvaniti V., D’Amico G., Fede G. (2010). Infections in patients with cirrhosis increase mortality four-fold and should be used in determining prognosis. Gastroenterology.

[7] D’Amico G., Garcia-Tsao G., Pagliaro L. (2006). Natural history and prognostic indicators of survival in cirrhosis: a systematic review of 118 studies. J Hepatol.

[8] Moreau R., Jalan R., Gines P. (2013). Acute-on-chronic liver failure is a distinct syndrome that develops in patients with acute decompensation of cirrhosis. Gastroenterology.

[9] Rothlin C.V., Ghosh S., Zuniga E.I. (2007). TAM receptors are pleiotropic inhibitors of the innate immune response. Cell.

[10] Lemke G., Rothlin C.V. (2008). Immunobiology of the TAM receptors. Nat Rev Immunol.

[11] Zagórska A., Través P.G., Lew E.D. (2014). Diversification of TAM receptor tyrosine kinase function. Nat Immunol.

[12] Fujimori T., Grabiec A.M., Kaur M. (2015). The Axl receptor tyrosine kinase is a discriminator of macrophage function in the inflamed lung. Mucosal Immunol.

[13] Deczkowska A., Keren-Shaul H., Weiner A. (2018). Disease-associated microglia: a universal immune sensor of neurodegeneration. Cell.

[14] Weinger J.G., Brosnan C.F., Loudig O. (2011). Loss of the receptor tyrosine kinase Axl leads to enhanced inflammation in the CNS and delayed removal of myelin debris during experimental autoimmune encephalomyelitis. J Neuroinflammation.

[15] Culemann S., Grüneboom A., Nicolás-Ávila J.Á. (2019). Locally renewing resident synovial macrophages provide a protective barrier for the joint. Nature.

[16] Bauer T., Zagórska A., Jurkin J. (2012). Identification of Axl as a downstream effector of TGF-β1 during Langerhans cell differentiation and epidermal homeostasis. J Exp Med.

[17] Zagórska A., Través P.G., Jiménez-García L. (2020). Differential regulation of hepatic physiology and injury by the TAM receptors Axl and Mer. Life Sci Alliance.

[18] Bernsmeier C., Pop O.T., Singanayagam A. (2015). Patients with acute-on-chronic liver failure have increased numbers of regulatory immune cells expressing the receptor tyrosine kinase MERTK. Gastroenterology.

[19] Triantafyllou E., Pop O.T., Possamai L.A. (2018). MerTK expressing hepatic macrophages promote the resolution of inflammation in acute liver failure. Gut.

[20] Brenig R., Pop O.T., Triantafyllou E. (2019). Expression of AXL receptor tyrosine kinase relates to monocyte dysfunction and severity of cirrhosis. Life Sci Alliance.

[21] Soulas C., Conerly C., Kim W.-K. (2011). Recently infiltrating MAC387+ monocytes/macrophages. Am J Pathol.

[22] Carpino G., Morini S., Ginanni Corradini S. (2005). Alpha-SMA expression in hepatic stellate cells and quantitative analysis of hepatic fibrosis in cirrhosis and in recurrent chronic hepatitis after liver transplantation. Dig Liver Dis.

[23] Mishra A., Wang J., Shiozawa Y. (2012). Hypoxia stabilizes GAS6/AXl signaling in metastatic prostate cancer. Mol Cancer Res.

[24] Lafdil F., Chobert M.N., Couchie D. (2006). Induction of Gas6 protein in CCl4-induced rat liver injury and anti-apoptotic effect on hepatic stellate cells. Hepatology.

[25] Guillot A., Winkler M., Silva Afonso M. (2023). Mapping the hepatic immune landscape identifies monocytic macrophages as key drivers of steatohepatitis and cholangiopathy progression. Hepatology.

[26] Ramachandran P., Dobie R., Wilson-Kanamori J.R. (2019). Resolving the fibrotic niche of human liver cirrhosis at single-cell level. Nature.

[27] Tutusaus A., de Gregorio E., Cucarull B. (2019). A functional role of GAS6/TAM in nonalcoholic steatohepatitis progression implicates AXL as therapeutic target. Cell Mol Gastroenterol Hepatol.

[28] Allavena P., Chieppa M., Monti P. (2004). From pattern recognition receptor to regulator of homeostasis: the double-faced macrophage mannose receptor. Crit Rev Immunol.

[29] Williams J.C., Craven R.R., Earp H.S. (2009). TAM receptors are dispensable in the phagocytosis and killing of bacteria. Cell Immunol.

[30] Remmerie A., Martens L., Thoné T. (2020). Osteopontin expression identifies a subset of recruited macrophages distinct from Kupffer cells in the fatty liver. Immunity.

[31] Bittmann I., Bottino A., Baretton G.B. (2003). The role of graft-resident Kupffer cells and lymphocytes of donor type during the time course after liver transplantation: a clinico-pathological study. Virchows Arch.

[32] Pallett L.J., Burton A.R., Amin O.E. (2020). Longevity and replenishment of human liver-resident memory T cells and mononuclear phagocytes. J Exp Med.

[33] Triantafyllou E., Woollard K.J., McPhail M.J.W. (2018). The role of monocytes and macrophages in acute and acute-on-chronic liver failure. Front Immunol.

[34] Kubes P., Jenne C. (2018). Immune responses in the liver. Annu Rev Immunol.

[35] Lu Q., Lemke G. (2001). Homeostatic regulation of the immune system by receptor tyrosine kinases of the Tyro 3 family. Science.

[36] Flint E., Triantafyllou E., Bernsmeier C. (2022). TAM receptors in the pathophysiology of liver disease. Livers.

[37] Bárcena C., Stefanovic M., Tutusaus A. (2015). Gas6/Axl pathway is activated in chronic liver disease and its targeting reduces fibrosis via hepatic stellate cell inactivation. J Hepatol.

[38] Cirera I., Bauer T.M., Navasa M. (2001). Bacterial translocation of enteric organisms in patients with cirrhosis. J Hepatol.

[39] Zapater P., Francés R., González-Navajas J.M. (2008). Serum and ascitic fluid bacterial DNA: a new independent prognostic factor in noninfected patients with cirrhosis. Hepatology.

[40] Schridde A., Bain C.C., Mayer J.U. (2017). Tissue-specific differentiation of colonic macrophages requires TGFβ receptor-mediated signaling. Mucosal Immunol.

[41] Du Plessis J., Vanheel H., Janssen C.E.I. (2013). Activated intestinal macrophages in patients with cirrhosis release NO and IL-6 that may disrupt intestinal barrier function. J Hepatol.

[42] Nishi C., Toda S., Segawa K. (2014). Tim4- and MerTK-mediated engulfment of apoptotic cells by mouse resident peritoneal macrophages. Mol Cell Biol.

[43] Ekman C., Linder A., Åkesson P. (2010). Plasma concentrations of Gas6 (growth arrest specific protein 6) and its soluble tyrosine kinase receptor sAxl in sepsis and systemic inflammatory response syndromes. Crit Care.

[44] Fourcot A., Couchie D., Chobert M.-N. (2011). Gas6 deficiency prevents liver inflammation, steatohepatitis, and fibrosis in mice. Am J Physiol Gastrointest Liver Physiol.

[45] Lee Y.A., Wallace M.C., Friedman S.L. (2015). Pathobiology of liver fibrosis: a translational success story. Gut.

[46] Di Blasi D., Boldanova T., Mori L. (2019). Unique T-cell populations define immune-inflamed hepatocellular carcinoma. Cell Mol Gastroenterol Hepatol.

[47] Van Gassen S., Callebaut B., Van Helden M.J. (2015). FlowSOM: using self-organizing maps for visualization and interpretation of cytometry data. Cytom Part J Int Soc Anal Cytol.

[48] Xu L. (2005). Human hepatic stellate cell lines, LX-1 and LX-2: new tools for analysis of hepatic fibrosis. Gut.

[49] Zimmermann H.W., Bruns T., Weston C.J. (2016). Bidirectional transendothelial migration of monocytes across hepatic sinusoidal endothelium shapes monocyte differentiation and regulates the balance between immunity and tolerance in liver. Hepatology.

[50] Triantafyllou E., Gudd C.L.C., Mawhin M.-A. (2021). PD-1 blockade improves Kupffer cell bacterial clearance in acute liver injury. J Clin Invest.

